# Evaluation of Reanalysis Surface Incident Solar Radiation Data in China

**DOI:** 10.1038/s41598-020-60460-1

**Published:** 2020-02-26

**Authors:** Xingxing Zhang, Ning Lu, Hou Jiang, Ling Yao

**Affiliations:** 1 State Key Laboratory of Resources and Environmental Information System, Institute of Geographic Sciences and Natural Resources Research, Chinese Academy of Sciences, Beijing, 100101 China; 2 College of Resources and Environment, University of Chinese Academy of Sciences, Beijing, 100049 China; 3 Jiangsu Center for Collaborative Innovation in Geographical Information Resource Development and Application, Nanjing, 210023 China

**Keywords:** Atmospheric science, Climate change, Energy access, Energy supply and demand

## Abstract

Surface incident solar radiation (R_s_) of reanalysis products is widely used in ecological conservation, agricultural production, civil engineering and various solar energy applications. It is of great importance to have a good knowledge of the uncertainty of reanalysis R_s_ products. In this study, we evaluated the R_s_ estimates from two representative global reanalysis (ERA-Interim and MERRA-2) using quality- controlled surface measurements from China Meteorological Administration (CMA) and Multi-layer Simulation and Data Assimilation Center of the Tibetan Plateau (DAM) from 2000 to 2009. Error causes are further analyzed in combination radiation products from the Earth’s Radiant Energy System (CERES) EBAF through time series estimation, hotspot selection and Geodetector methods. Both the ERA-Interim and MERRA-2 products overestimate the R_s_ in China, and the MERRA-2 overestimation is more pronounced. The errors of the ERA-Interim are greater in spring and winter, while that of the MERRA-2 are almost the same in all seasons. As more quality-controlled measurements were used for validation, the conclusions seem more reliable, thereby providing scientific reference for rational use of these datasets. It was also found that the main causes of errors are the cloud coverage in the southeast coastal area, aerosol optical depth (AOD) and water vapor content in the Sichuan Basin, and cloud coverage and AOD in the northeast and middle east of China.

## Introduction

Surface incident solar radiation (R_s_) is the basic energy of biological, physical and chemical processes, and the essential input parameters of biological physics models and hydrological simulation mathematical models^1,2^. Ground-based stations provide the best estimate of R_s_, but it is still insufficient for estimation in remote areas, especially in high latitudes, and in plateaus or mountainous areas, due to the sparsity and heterogeneity of stations^3–6^. Currently, there exists a range of gridded global R_s_ products with higher spatial resolution exist from remote sensing^7,8^ and reanalysis^9,10^. Satellite remote sensing is one of the most practical ways to derive R_s_ with relatively higher accuracy, but temporal coverage is limited by transit time of satellite^11,12^.

In addition to satellite-based products, scientists have been developing reanalysis R_s_ products to restore long-term historical climate records for numerical weather forecasting using data assimilation techniques since the late 1980s^13^. Reanalysis methods provide dynamically consistent global analysis of the global atmospheric characteristics by combining the geophysical fluid-dynamic model of the atmosphere and measurements. The model contains important physical processes, such as radiative transfer and convection. Observations are used to constrain the dynamic model to optimize the properties of complete coverage and accuracy^14^. Reanalysis data provides global and effective R_s_ of long time series, which alleviate the deficiency in radiation data and greatly promote the development of modern atmospheric science. As reanalysis data are obtained by numerical simulation, they cannot completely replace the observed data for describing the real three-dimensional state of the atmosphere^15^. Due to the heterogeneity of various data sources and difference of data assimilation schemes, there exist errors in radiation reanalysis products^16^. Thus, understanding the uncertainty and deviation of reanalysis data is a prerequisite for the rational use of reanalysis data^17^.

Since the mid-1990s, the United States, European Union, and Japan have organized and implemented a series of global reanalysis projects on atmospheric data to restore and reconstruct historical records of climate change. There are six representative reanalysis products: ERA-Interim of the European Centre for Medium-Range Weather Forecasts (ECMWF); MERRA-2 of the National Aeronautics and Space Administration (NASA) Goddard Space Flight Center (GSFC)’s Global Modeling and Assimilation Office (GMAO); NCEP–NCAR reanalysis, NCEP-DOE reanalysis, Climate Forecast System Reanalysis (CFSR) from the National Centers for Environmental Prediction (NCEP); JRA-55 from the Japan Meteorological Agency (JMA). These reanalysis data are widely used in the field of atmospheric sciences, for example, surface temperature changes, regional precipitation distribution, and surface solar radiation distribution^18–20^. Among them, reanalysis R_s_ products are widely used in ecological conservation, agricultural production, and civil engineering and various solar energy applications^21–23^.

In recent decades, multi-source R_s_ data such as ground measurements and satellite inversed products, have been used to perform multi-scale verification of reanalysis R_s_ data at stations, regions, or on a global scale. It shows that the R_s_ of reanalysis data is usually larger than ground measurements in most cases^24–27^. Wang and Zeng^24^ found that both the MERRA and ERA-Interim slightly overestimate R_s_ by 1.56–5.00 W/m^2^ on a daily scale based on nine stations from the Coordinated Enhanced Observing Period (CEOP)-Asia-Australia Monsoon Project (CAMP/Tibet). Yue^28^ found that the annual average of R_s_ from MERRA is 3–4 W/m^2^ higher than the observed value in the Yangtze River Delta region, but their inter-annual changes are basically the same. Yue^28^ further analyzed the influence of aerosol content on the R_s_ of MERRA. Fu *et al*.^29^ assessed the applicability of four reanalysis data (NCEP-1, NCEP-2, ERA-Interim and JCDAS) in the southeast polar region by using the radiative observation data of the Panda-1 station from February 2011 to January 2012. The results showed that the ERA-Interim is state-of-the-art, and its average deviation (*bias*) is −22.70 W/m^2^. The deviation is small in summer (4.82 W/m^2^) and large in winter (24.70 W/m^2^). The applicability of reanalysis data is distinctly different in different stations, different regions or different periods. However, above studies are limited by the number of stations. Thus, more observation data are needed to confirm the applicability of reanalysis R_s_. Zhang *et al*.^17^ used 674 ground-based observation stations to conduct research on the seasonal changes and spatial distribution at the global scale taking advantage of six kinds of reanalysis R_s_ products (NCEP–NCAR, NCEP-DOE, CFSR, ERA-Interim, MERRA, and JRA-55). The study showed that all products overestimate R_s_ in China, and the monthly difference between reanalysis R_s_ and measurements ranges from 23.15 to 71.95 W/m^2^. ERA-Interim and MERRA have relatively better quality in China compared with other products, and the data quality shows obvious seasonal differences. Zhang *et al*.^17^ preliminarily discussed that the underestimation of cloud coverage on the global scale may lead to the overestimation of reanalysis R_s_. Boilley and Wald^30^ compared the meteorological reanalysis from ERA-Interim and MERRA and measurements of daily solar irradiation on surface in Europe, Africa and Atlantic Ocean, which proved that the reanalysis often mistakes cloudy conditions as clear skies. Penna *et al*.^31^ demonstrated that AOD could cause a difference of R_s_ up to 30 W/m^2^ over the Amazon region in MERRA-2, which was associated with the absorption of aerosols. In fact, solar radiation varies on spatial-temporal scales and influenced by many factors, such as cloud coverage, AOD, water vapor content, ozone concentration, surface albedo and other factors. Previous studies have concentrated on the evaluation of reanalysis R_s_ but ignored the causes of the errors or only consider a single factor in a large region. It is vital to have a good knowledge of the main impact factors in the different seasons and regions for the purpose of proper application and accurate data correction.

In this paper, we analyze the spatial-temporal errors of the MERRA-2 and ERA-Interim in China and identify the causes of the errors. The observation station data from the China Meteorological Administration (CMA) and Multi-layer Simulation and Data Assimilation Center of the Tibetan Plateau (DAM) are used to assess R_s_ of MERRA-2 and ERA-Interim. Meanwhile, the MERRA-2 and ERA-Interim products are compared to determine the difference in their spatial distributions and seasonal variations. The Clouds and Earth’s Radiant Energy Systems (CERES) Energy Balanced and Filled (EBAF) R_s_ dataset^8^ is used to identify regions with large radiation deviations in different seasons as hotspots through comparison to reanalysis radiation data. Considering the influence of atmospheric factors on the reanalysis R_s_ products and the spatial heterogeneity of the distribution of atmospheric factors, we introduce the Geodetector^32^ to quantitatively analyze the causes of the spatial-temporal errors of R_s_ in the hotspots and utilize CERES-EBAF atmospheric products to verify the results of the dominant atmospheric influence factors. The results are useful for the proper application and accurate data correction about the two representative global reanalysis data.

This paper is organized as follows. The reanalysis products (ERA-Interim and MERRA-2) and station measurements (CMA and DAM stations) used for this study region, the description of the quality control of the stations and the rationale of using the Geodetector are detailed in Section 2. The errors of reanalysis are validated, and the domain impact factor based on the Geodetector is analyzed in Section 3. A short summary and conclusions are presented in Section 4.

## Experiments

### Data

Five R_s_ data sources are used in this study: two reanalysis products, one satellite remote sensing based product, one ground measurement and one model simulation dataset.

#### Reanalysis products

Two reanalysis products, ERA-Interim and MERRA-2, are used in this study. These products are different in many aspects, such as physical parameterizations of numerical models, numerical schemes, observational data used for assimilation, and the assimilation schemes^17,24^. The monthly mean R_s_ data of these two reanalysis datasets from 2000 to 2009 are evaluated in this research. The details of these datasets are described in the following paragraphs.

The ERA-Interim^9^ is provided by the European Centre for Medium Range Weather Forecasts (ECMWF), which is one of the most important reanalysis centers in the world. The spatial resolution of the ERA-Interim is 0.75° (approximately 80 km) in the horizontal direction, which is divided into 60 levels from the ground surface to the 0.1hPa altitude in the vertical direction. The temporal resolution of the ERA-Interim is 3 h. An improved 3DVar assimilation technology is used as the assimilation scheme. The data sources used in the assimilation scheme include ground measurements and satellite remote sensing data. The radiation scheme is based on the Rapid Radiation Transfer Model (RRTM)^33^. The prognostic cloud variables (cloud cover, cloud condensed water) and water vapor from the meteorological model and climatologic values for aerosols, carbon dioxide, trace gases and ozone are used in the radiative transfer model^30,34^.

The MERRA-2^10^ is provided by the GSFC’s Global Modeling and Assimilation Office (GMAO). It was introduced to replace the original MERRA dataset because of the advances made in the assimilation system that enable assimilation of modern hyperspectral radiance and microwave observations, along with GPS-Radio Occultation datasets. It also uses NASA’s ozone profile observations that began in late 2004. Additional advances in both the GEOS (Goddard Earth Observing System Data Assimilation System) model and the GSI (Gridpoint Statistical Interpolation) assimilation system are included in MERRA-2. Spatial resolution remains about 50 km in the latitudinal direction. Along with the enhancements in the meteorological assimilation, MERRA-2 takes some significant steps towards GMAO’s target of an Earth System reanalysis. The radiation scheme is based on the method proposed by Chou and Suarez^35^. In the MERRA-2 reanalysis, the meteorological and aerosol observations are simultaneously assimilated within the GEOS-5. The MODIS Neural Net Retrieval and AVHRR Neural Net Retrieval at 550 nm are used to assimilate the AOD observations^30^.

#### CMA ground measurements

**Figure 1:**
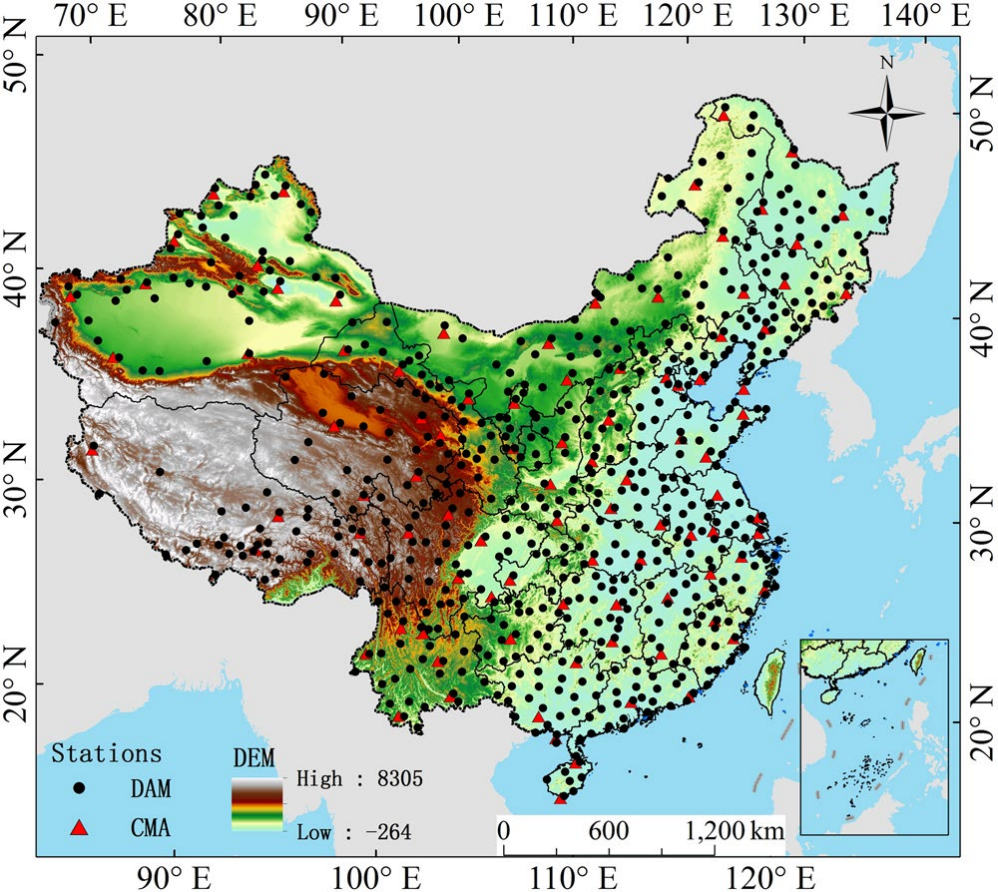
Geographical distribution of the observation stations (812 stations in total) used in this study from CMA (96 stations in red triangles), and DAM (716 stations in black dots). (Generated by Arcgis 10.7 software, https://www.esri.com/en-us/home).

The CMA provides ground radiation measurements of 122 stations in China (except Taiwan, Hong Kong, and Macao). The CMA surface observations include three basic physical variables: the total surface solar radiation R_s_, direct solar radiation R_dir_, and diffuse radiation R_dif_ from 1957 to the present. The instruments of stations used are: DFY-4 and TBQ-2 total radiation meter; DFY-3 and TBS-2 direct radiation meter (both with solar tracking frame); DFP-1 shading ring; and RYJ-4 automatic radiation recorder. The above-mentioned radiation meters are all electrothermal type, which consists of two parts: the induction surface and the thermopile^36,37^. The radiation meter and measuring instrument (voltmeter, ammeter) constitute a set of radiation instruments. Detail information of instruments for CMA stations are available on the website: http://data.cma.cn/site/index.html. The world radiation reference (WRR) is used as the standard to carry out the value transmission in these stations, ensuring the comparability of solar radiation measurements worldwide. The distribution of these radiation stations is shown in Fig. 1 (red triangles).

#### Daily surface solar radiation dataset of the DAM

Tang *et al*.^38–40^ developed hybrid model and daily meteorological data (including air temperature, atmospheric pressure, relative humidity, sunshine duration hours, and precipitation) to estimate the daily R_s_ of the 716 CMA meteorological stations based on published data from the 716 regular meteorological stations of the Weather Information Centre of the CMA. Neural network models are built on each CMA 96 radiation station, and then the trained models are used to estimate the R_s_ of the 716 CMA stations. Finally, the estimation results of the artificial neural network model are used to correct those of the hybrid model for the purpose of obtaining 716 daily R_s_ data in China from 1961 to 2010 (http://www.tpedatabase.cn/portal/MetaDataInfo.jsp?MetaDataId=249399). The sparse and uneven spatial distribution of CMA stations is compensated in this way. The distribution of the radiation stations is also shown in Fig. 1 (black circles).

#### CERES-EBAF product

The CERES-EBAF product is derived from the clouds, AOD, and earth radiant energy systems detected by the TRMM, Terra and Aqua satellites. It provides a reasonable inversion of atmospheric parameters such as clouds, water vapor and AOD and R_s_ estimation. Atmospheric parameters are derived from A-train Constellation (the Cloud Aerosol Lidar with Orthogonal Polarization (CALIOP) on the Cloud Aerosol Lidar and Infrared Pathfinder Satellite Observation (CALIPSO) satellite, the CloudSat Cloud Profiling Radar (CPR), and the Aqua Moderate Resolution Imaging Spectrometer (MODIS)). R_s_ are derived from a radiative transfer model of CERES with k-distribution and correlated-k for radiation (FLCKKR) with a two stream approximation, which is consistent with the radiative flux from the surface to the top of the atmosphere^41^. The CERES-EBAF product contains 1° regional, zonal and global monthly means of Top-of-Atmosphere (TOA) and surface (SFC) longwave (LW), shortwave (SW), and net (NET) fluxes under clear and all-sky conditions. The CERES-EBAF has higher accuracy than the other grid R_s_ products like CMIP5, NCEP-NCAR, NCEP-DOE, CFSR, ERA-Interim, MERRA and JRA-55 *et al*.^17,41,42^. Zhang *et al*.^17^ found that the CERES-EBAF R_s_ data are more consistent with ground measurements than the reanalysis data, and provide more accurate atmospheric products such as clouds and aerosols. Li *et al*.^43^ proved that AODs used by CERES have mean *biases* less than 0.1 and small Root Mean Square Error (*RMSE*) over most sites in China compared with China Remote Sensing Network (CARSNET). Yan *et al*.^44^ found that the time series of CERES cloud properties, for the most part, closely track the variations of the surface retrievals and their correlation coefficient reaches up to 0.83. Liu *et al*.^45^ evaluated the water vapor product of MODIS at the 83 stations in China. The results show that the water vapor has high correlation coefficient (more than 0.91) with station measurements, yielding a mean *bias* and *RMSE* less than 2.58 mm and 6.02 mm respectively. The EBAF R_s_, AOD, Cloud Coverage and Water Vapor Content of CERES are used in this study. Due to the lack of higher spatiotemporal resolution ground measurements, in this paper, the CERES data were averaged in a long time series for subsequent analysis and comparisons, which greatly reduced the uncertainty of the data and enhanced the credibility of the experiment.

### Methodology

#### Station quality control

There are some quality problems in the CMA ground measurements, resulting from the accuracy and calibration of radiation instruments, the human factors in the operation of instruments, and the positional changes in stations^46^. When using CMA radiation data as validation data, a quality inspection should be carried out first. The original station data have been checked by a simple physical quality control of the daily R_s_ data; that is, the R_s_ is equal to the sum of diffuse radiation and the direct radiation (Original threshold control: ${R}_{s}={R}_{dif}+{R}_{dir}$). Furthermore, an upper limit is set for further validation. Referring to Shi’s research^37^, the upper limit is the solar radiation *G*_0_ received by the earth’s top atmosphere per day:

A1 $${G}_{0}=\frac{24\times 3600}{\pi }{I}_{0}k({\rm{c}}{\rm{o}}{\rm{s}}\varphi {\rm{c}}{\rm{o}}{\rm{s}}\delta {\rm{s}}{\rm{i}}{\rm{n}}{w}_{s}+\frac{\pi {w}_{s}}{180}\,\sin \,\varphi \,\sin \,\delta )$$

where *I*_0_ is the solar constant (approximately 1367 W/m^2^), *δ* is the solar declination angle, *φ* is the station latitude, *w*_*s*_ is the sunset angle, and *k* is the earth orbit eccentricity correction factor. The daily earth orbit eccentricity correction factor *k* is calculated using the following formula^47^:

A2 $$k=1+0.033{\rm{c}}{\rm{o}}{\rm{s}}(\frac{360{d}_{n}}{365})$$

where *k* is the ordinal day of the year and *d*_*n*_ represents the day of the year. The daily solar declination angle *δ* and sunset angle *w*_*s*_ are calculated using the following formulas^48,49^:

A3 $$\delta =23{\rm{.45sin}}(360\frac{284+{d}_{n}}{365})$$

A4 $${w}_{{\rm{s}}}={\rm{c}}{\rm{o}}{{\rm{s}}}^{-1}(-\,\tan \,\phi \,\tan \,\delta )$$

From a physical point of view, it is difficult to set a strict lower limit for the R_s_. Due to the existence of scattered radiation; the R_s_ must be greater than zero. Geiger *et al*.^50^ based on the statistical characteristics of the radiation data, the lower limit is set to 3% of the daily solar radiation received by the top of the earth’s atmosphere every day (see Eq. (A5)). Such simple quality control is proved to be effective can eliminate most of the error^51–54^. Moreover, CMA data is checked for errors at daily scale. The final used data is the monthly average data calculated from daily R_s_. Table 1 summarizes the results of the physical threshold test. From the table, we can see that the incidence of errors is lower in summer. The average error rate of the daily R_s_ observations from 2000 to 2009 is 1.98%, and the winter error rate is higher than that of the summer. In summary, only ground measurements that pass the following physical criterion are used for validation.

A5 $$0.03\ast {G}_{0} < {R}_{s} < {G}_{0}$$

**Table 1:** Monthly distribution of the number of days for daily R_s_ data of CMA failing the physical threshold test from 2000 to 2009.

	Month												Total
	1	2	3	4	5	6	7	8	9	10	11	12	
Upper-limit error days	75	60	216	307	477	330	276	171	123	138	100	55	2328
Lower-limit error days	695	516	397	305	323	298	201	259	364	436	510	539	4843
Passing test days	29920	27569	30077	29088	29890	29072	30213	30260	29213	30116	29090	30096	354604
Percent of error days (%)	2.51	2.29	2.00	2.06	2.61	2.11	1.55	1.40	1.64	1.87	2.05	1.94	1.98

Considering that the CMA stations are sparse and unevenly distributed, the 716 R_s_ data of the DAM is also used in this study. The daily surface solar radiation dataset was produced by merging two data sets. One is the hybrid model estimate at 716 CMA stations and the other is the ANN-based model estimate at 96 radiation stations. The latter, which has higher accuracy, was used to correct the hybrid model estimate dynamically at a monthly scale. Tang *et al*.^39,40^ verified the accuracy of the assimilated radiation data set And showed that the *bias* and *RMSE* of the hybrid model at the 96 CMA radiation stations are 0.7 and 2 MJ/m^2^ respectively, While that of the corrected hybrid model are −0.1 and 1.8 MJ/m^2^ respectively. The accuracy of the corrected radiation dataset is significantly higher than that of the traditional locally calibrated model. Therefore, the 716 R_s_ data can be used as valid data for evaluating the reanalysis data. In this study, both CMA and DAM are used to evaluate the reanalysis products for a better comparison. But only DAM is used to analyze the error causes of reanalysis because it is highly consistent with CMA and has more data than CMA.

#### Time series estimation

In this study, the R_s_ data from 2000 to 2009 by ERA-Interim and MERRA-2 are compared with the CMA and DAM measurements, and the data error of reanalysis at different time scales is analyzed. The daily R_s_ estimates of ERA-Interim and MERRA-2 are horizontally interpolated using a bilinear interpolation technique with inverse distance weights for four most closest surrounding grid cells^30^.

To investigate the error distribution of the reanalysis data at the different stations, the seasonal distribution of the average relative bias (*RB*) for the reanalysis and measured R_s_ from 2000 to 2009 is calculated. The expression of the *RB* is as follows:

A6 $$RB=\frac{{R}_{s\_Reanalysis}-{R}_{s\_sites}}{{R}_{s\_sites}}\ast 100$$

where ${R}_{s\_Reanalysis}$ and ${R}_{s\_sites}$ represent the R_s_ of reanalysis products and stations, respectively. Where *RB* indicates relative bias, and positive values, indicate that the reanalysis data is higher than the ground observation data, while negative values indicate that the reanalysis data is lower than the ground observation data.

The expression of the monthly anomalies is as follows:

A7 $$D{A}_{i}=SS{R}_{i}-\overline{SSR}$$

where *SSR*_*i*_ and $\overline{SSR}$ are the average monthly R_s_ and the average monthly R_s_ for all years of that month, respectively.

The expression of the *RMSE* is as follows:

A8 $$RMSE=\sqrt{\frac{1}{m}{\mathop{\sum }\limits_{i=1}^{m}({R}_{s\_\mathrm{Re}analysis}-{R}_{s\_sites})}^{2}}$$

where *m* is the numbers of month.

#### Geodetector

The Geodetector is a method used to explore the influence mechanism of geographical spatial zoning factors on disease risk in infancy^55^. It can effectively identify the relationship between multiple factors and geographical phenomena, so it has been gradually applied to the study of geography and humanities^56,57^. The factor detector of the Geodetector can verify the spatial heterogeneity of a single variable. Atmospheric factors such as AOD, cloud coverage and water vapor content are typical category variables and have an important influence on the reanalysis of R_s_. Therefore, it is suitable to use the Geodetector method to reveal the influence of the regional atmospheric factors on the reanalysis of the surface radiative error. The Geodetector software is divided into four parts: the risk detector (superposition of the related data, then comparison of whether the difference is significant, and the major role of the significant factor in the risk is determined), the factor detector (using the q value^55^ to test the association between two variables Y and X, according to the coupling between their spatial distributions, without assumption of linearity), the ecological detector (using the variance to compare), and the interaction detectors (including synergy, antagonism, double synergy, single antagonism and mutual independence). The factor detector section is used in this study.

**Figure 2:**
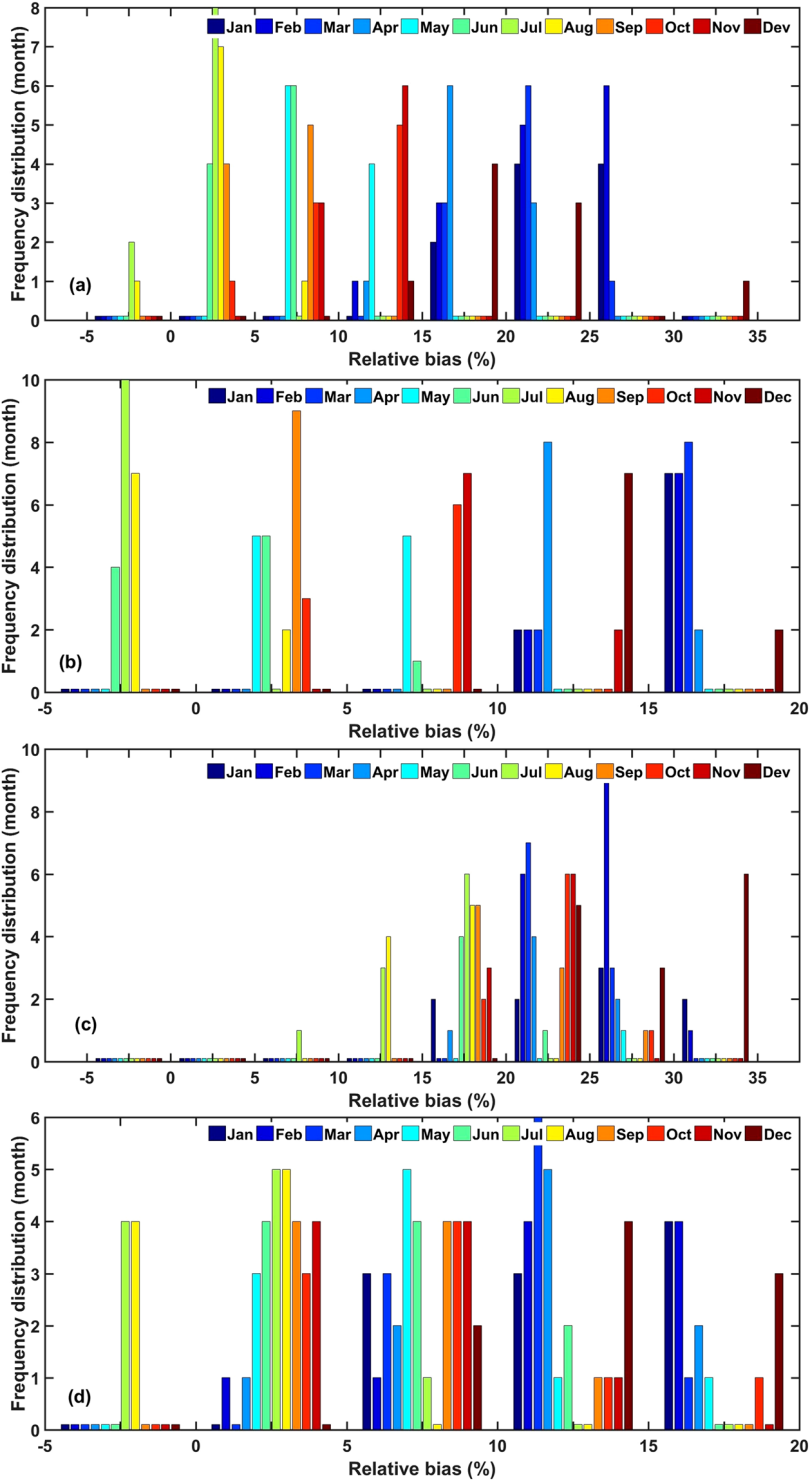
*RB* frequency distribution of reanalysis radiation products (ERA-Interim and MERRA-2) and ground measurements (CMA and DAM) from 2000 to 2009, (**a**,**b**) represent the *RB* frequency distribution of ERA-Interim and CMA or DAM in different months, respectively. (**c**,**d**) represent the *RB* frequency distribution of MERRA-2 and CMA or DAM in different months, respectively. The vertical axis of frequency distribution (month) represents the numbers of month which *RB* is in the classification range of the horizontal axis.

**Figure 3:**
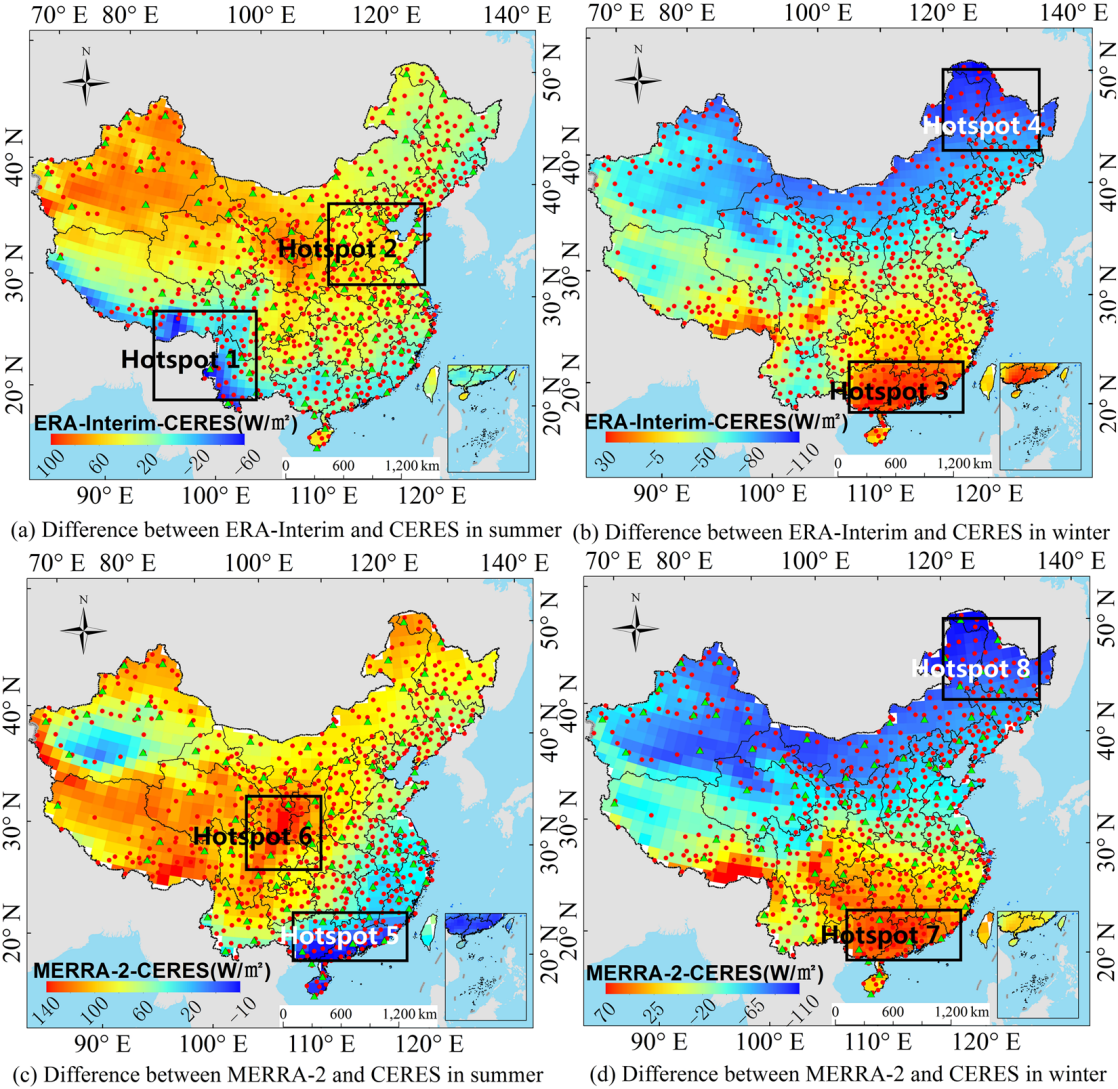
The average R_s_ hotspot regions’ selection of ERA-Interim and MERRA-2 from 2000 to 2009 ((**a**,**b**) represent ERA-Interim’s hotspot regions in summer and winter, respectively. (**c**,**d**) represent MERRA-2’s hotspot regions in summer and winter, respectively.). (Generated by Arcgis 10.7 software, https://www.esri.com/en-us/home).

To analyze the significance of the atmospheric influence factors on the R_s_ errors in the different regions of China, we actually analyze 10 factors at first, including the reanalysis ozone concentration, surface albedo, content of ice and water clouds, cloud coverage, AOD and water vapor content, etc. It is found that the influence of AOD, cloud coverage and water vapor content on the R_s_ is obvious, while other factors are not significant. This is basically consistent with the results of sensitivity analysis of radiation transmission models^47^. Therefore, this paper focuses on the quantitative influence level of AOD, cloud coverage and water vapor content on the R_s_ error in the different regions. Taking the *RB* of R_s_ at each regional station as the spatial stratification variable *Y*, the influence of atmospheric factors on the *RB* of reanalysis R_s_ is measured based on the power of determinant (*PD*) value of the Geodetector model. The region used to analyze the cause of error must satisfy the following conditions: the station distribution in the region is even and as many stations as possible, and error analysis is conducted on the region where the reanalysis R_s_ deviation is large. These regions are called hotspots in this study. Studies^3,8,17,42^ have shown that CERES-EBAF has a higher accuracy than the other grid R_s_ products; thus, the CERES-EBAF R_s_ data are used as verification data. Zhang *et al*.^17^ proved that almost all the reanalysis R_s_ products showed better accuracy in summer (June, July, and August) than in winter (December, January, and February), and the difference between summer and winter is more prominent than other seasons. Section 3.1 of this study further confirmed that both ERA-Interim and MERRA-2 have lager frequency distribution with smaller *RB* in summer than winter (Fig. 2). So, the reanalysis monthly mean R_s_ products were divided into summer and winter seasons to assess the seasonal error causes (Fig. 3). To perform the comparison, a bilinear interpolation of a weighted average of pixel in the nearest 2-by-2 neighborhood was used to unify the resolution of the reanalysis products to CERES-EBAF. According to the map, this study selects the large deviation regions of the reanalysis R_s_ in summer and winter as a hotspot area. Two hotspots in each season are selected, and a total of 8 hotspots are numbered 1–8. For the ERA-Interim, the southwestern region (hotspot 1) and the eastern region (hotspot 2) are selected as the hotspots during the summer, while the southern coastal region (hotspot 3) and the northeastern region (hotspot 4) are selected in winter. For the MERRA-2, the southern coastal region (hotspot 5) and the Sichuan Basin region (hotspot 6) are selected in summer, and the southern coastal region (hotspot 7) and the north-eastern region (hotspot 8) are selected in winter. The same region but different hotspot numbers was defined when the seasons or reanalysis product are different for better comparison, such as the hotspots 3, 5 and 7, and the hotspots 4 and 8.

**Figure 4:**
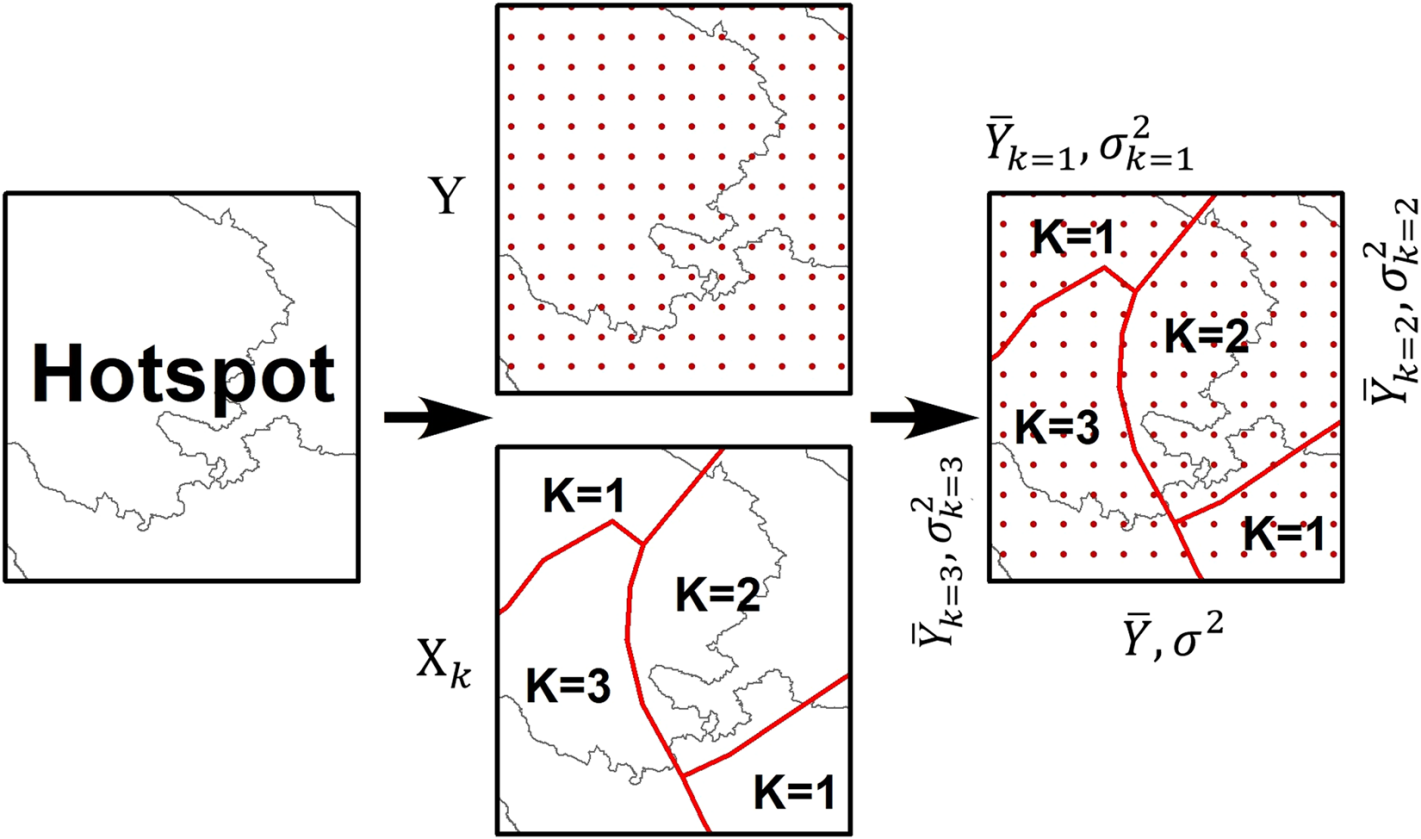
The principle of the geographical detector.

Based on the reanalysis seasonal differences, we use Geodetector to analyze the causes of the errors in the hotspots. As shown in Fig. 4, K-means clustering analysis is first used to divide the hotspot into *k* sub-regions with atmospheric factor *X*. The number of R_s_ stations and the variance of the *RB* of the R_s_ of the stations in the sub-regions are recorded as ${N}_{d1},\,{N}_{d2},\ldots ,{N}_{dk}$ and ${\sigma }_{d1}^{2},\,{\sigma }_{d2}^{2},\ldots ,{\sigma }_{di}^{2}$ respectively.

A9 $$PD=1-\frac{{\sum }_{i=1}^{{\rm{K}}}({N}_{di}\times {\sigma }_{di}^{2})}{{N}_{D}\times {\sigma }_{D}^{2}}$$

where *i=*1, 2, 3, …, *k*, represents the number of sub-regions; *N*_*di*_ is the number of stations in the sub-region of the *i*; *N*_*D*_ is the number of stations in the entire hotspot, ${N}_{D}={\sum }_{i=1}^{{\rm{K}}}({N}_{di})$; and ${\sigma }_{D}^{2}$ represents the discrete variance of the *RB* of the R_s_ over the whole hotspot’s station. The study area is divided into *k* sub-regions based on atmospheric factors. When the atmospheric factor *X* has a decisive force on the *RB* of the station, the gap between the atmospheric factors in each sub-region is small while the gap between the sub-regions is large. The discrete variance ${\sigma }_{di}^{2}\,$ of the station is small in each sub-region, while the variance between the sub-regions is very large. When ${\sigma }_{di}^{2}$ is close to 0, the *PD* value tends to be 1, which is the ideal state indicating that the *RB* changes are completely determined by atmospheric factor *X*. If the *RB* of the R_s_ is irrelevant to the atmospheric factor, then the weighted sum of the discrete variance ${\sigma }_{di}^{2}$ and the number of stations in each sub-region *N*_*di*_ is closer to *VarD* $(VarD={N}_{D}\times {\sigma }_{D}^{2})$, thus *PD* = 0. Therefore, the range of the *PD* value is [0, 1]. A larger *PD* value indicates a greater correlation between the reanalysis atmospheric factors and *RB* of the reanalysis R_s_.

## Results and Discussion

### Correlation and trend analysis

**Figure 5:**
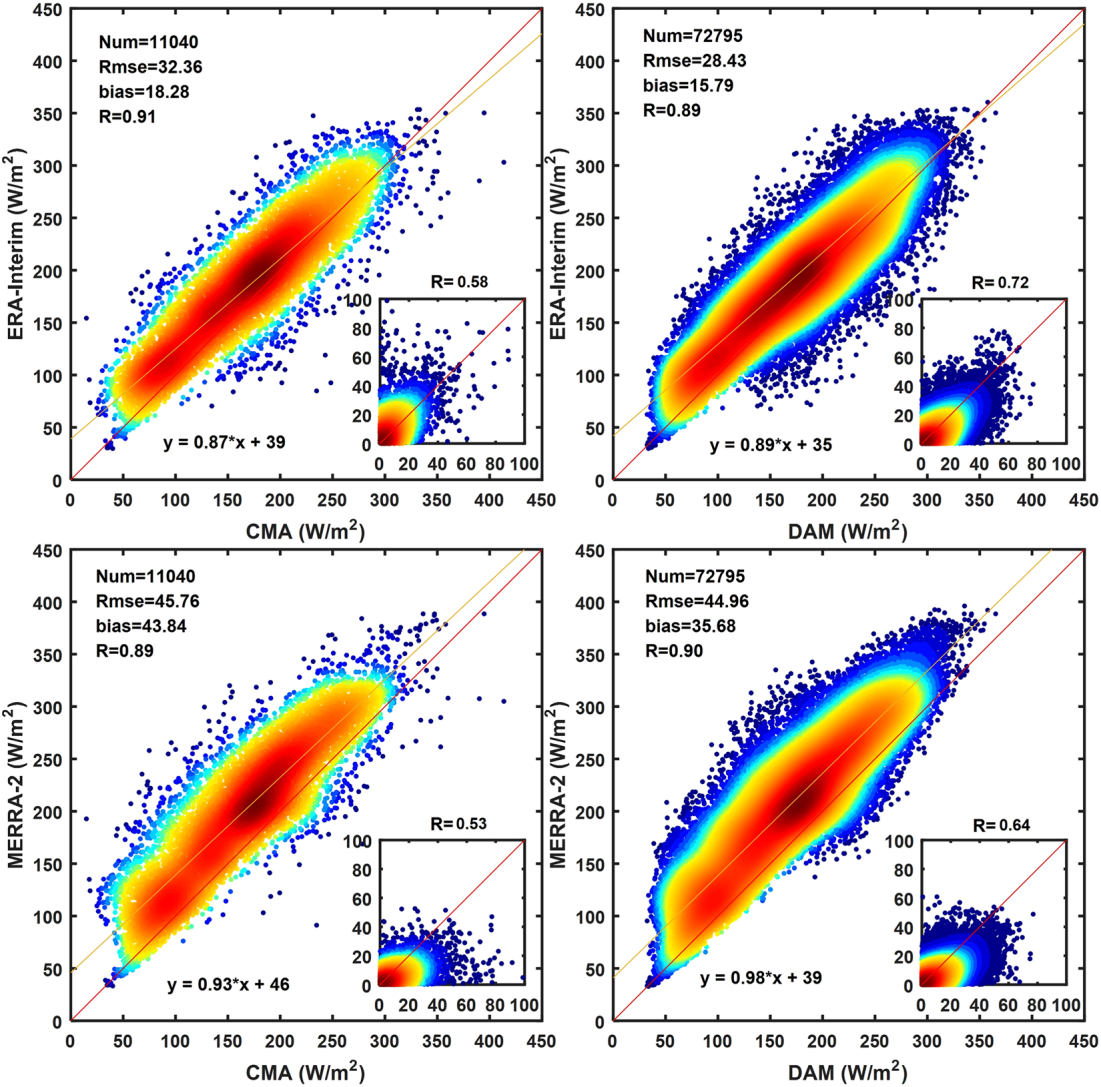
Average monthly R_s_ scatter plots between reanalysis products (ERA-Interim and MERRA-2) and surface measurements (CMA and DAM) from 2000 to 2009. (The color code represents the dot density, and the dot density in the red area is higher than that in the blue area. Deseasonalized correlations in the lower right corner.).

Figure 5 shows the scatter plots of monthly average surface radiation for the reanalysis and stations (CMA and DAM) from 2000 to 2009. The correlation coefficient (*R*) between ERA-Interim and station measurements is 0.91 and 0.89 respectively; the *RMSE* values are 32.36 and 28.43 W/m^2^ respectively; and the *bias* is 18.28 and 15.79 W/m^2^, respectively. The *R* between the MERRA-2 and stations is 0.93 and 0.98, respectively; the *RMSE* is 45.76 and 44.96 W/m^2^, respectively; and *bias* is 43.84 and 35.68 W/m^2^, respectively. The difference between the two reanalysis products and CMA or DAM is very small; the difference of *RMSE* is 3.93 and 0.80 W/m^2^, respectively; and the difference of the *bias* is 2.49 and 8.16 W/m^2^, respectively, which further illustrates that the DAM datasets have high consistency with CMA and can be used as validation data. In addition, the experiment shows that the reanalysis of the R_s_ data by ERA-Interim and MERRA-2 is higher than the ground observation station data, which is consistent with the previous conclusions^17,58,59^. The *biases* of the ERA-Interim and MERRA-2 are 15.79–18.28 W/m^2^ and 35.68–43.84 W/m^2^, respectively, and the *RMSEs* are 28.43–32.36 W/m_2_ and 44.96–45.76 W/m^2^, respectively. In general, the R_s_ in china is overestimated by both reanalysis products, but the overestimation by MERRA-2 is more obvious.

**Figure 6:**
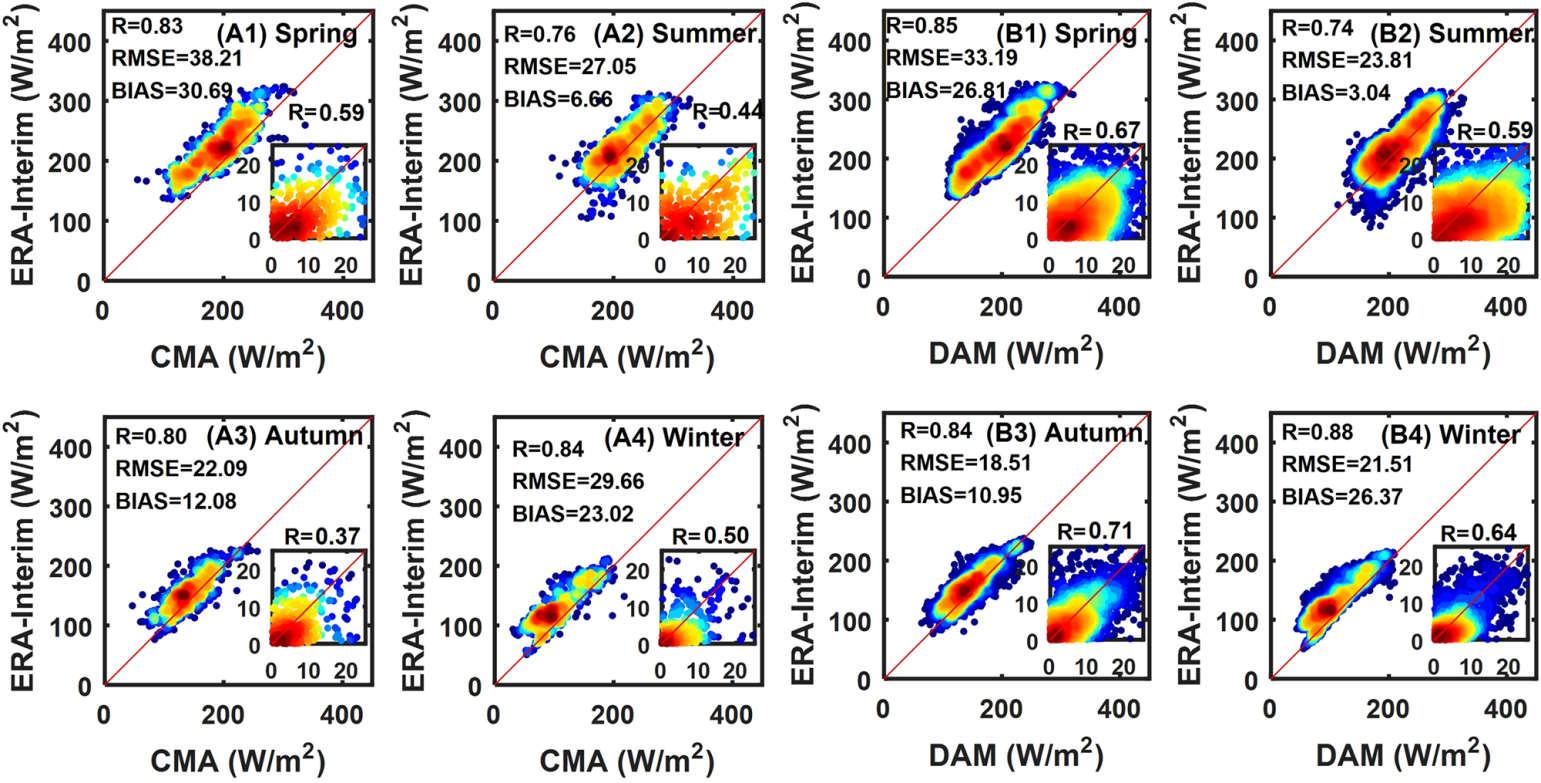
Seasonal average monthly R_s_ scatter plots between ERA-Interim and surface measurements (CMA and DAM) from 2000 to 2009. ((A1) – (A4) represent the scatter plots of the average monthly R_s_ of ERA-Interim and CMA in different seasons. (B1) – (B4) represent the scatter plots of average monthly R_s_ of ERA-Interim and DAM in different seasons. The color code represents the dot density, and the dot density in the red area is higher than that in the blue area. Deseasonalized correlations in the lower right corner.).

**Figure 7:**
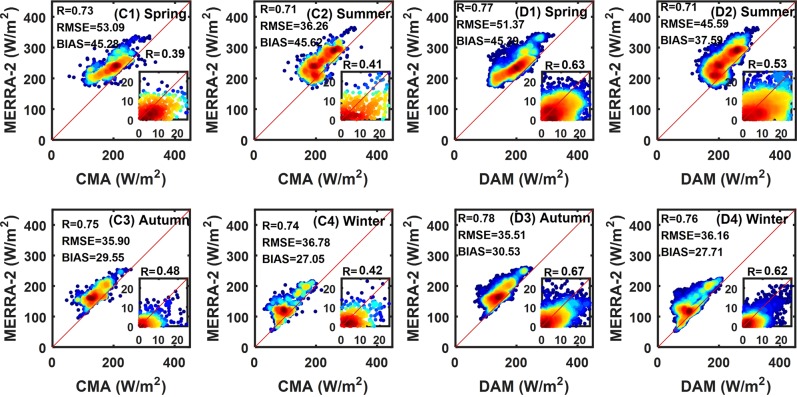
Seasonal average monthly R_s_ scatter plots between MERRA-2 and surface measurements (CMA and DAM) from 2000 to 2009. ((C1) – (C4) represent the scatter plots of average monthly R_s_ of MERRA-2 and CMA in different seasons. (D1) – (D4) represent the scatter plots of average monthly R_s_ of MERRA-2 and DAM in different seasons. The color code represents the dot density, and the dot density in the red area is higher than that in the blue area. Deseasonalized correlations in the lower right corner.).

Reanalysis shows seasonal differences (Table 2), and Figs. 6 to 7 show the seasonal scatter plots of the reanalysis with the stations from 2000 to 2009. The ERA-Interim shows obvious seasonal differences under the verification of CMA and DAM. The ranges of *bias* and *RMSE* in summer and autumn are 3.04–12.08 W/m^2^ and 18.51–27.05 W/m^2^, respectively, but are 23.02–38.21 W/m^2^ and 21.51 W/m^2^–38.21 in spring and winter, respectively, which is higher than that in summer and autumn season. The seasonal characteristics of the MERRA-2 are not obvious, and the range of *bias* in summer and autumn is 29.55–45.62 W/m^2^, which close to that in spring and winter. However, the range of the *RMSE* in spring and winter is 35.59–53.09 W/m^2^, which is slightly higher than that in summer and autumn.

**Table 2:** Evaluation of the seasonal R_s_ from the ERA-Interim and MERRA-2 products using surface measurements collected from the CMA and DAM stations from 2000 to 2009.

Reanalysis	ERA-Interim						MERRA-2					
	Bias		RMSE		R		Bias		RMSE		R	
Season	CMA	DAM	CMA	DAM	CMA	DAM	CMA	DAM	CMA	DAM	CMA	DAM
Spring	30.69	26.81	38.21	33.19	0.83	0.85	45.28	45.39	53.09	51.37	0.73	0.77
	0	0	13.33	8.78	0.59	0.67	0	0	15.16	8.75	0.39	0.63
Summer	6.66	3.04	27.05	23.81	0.76	0.74	45.62	37.59	36.26	45.59	0.71	0.71
	0	0	14.82	11.03	0.44	0.59	0	0	14.72	11.41	0.41	0.53
Autumn	12.08	10.95	22.09	18.51	0.80	0.84	29.55	30.53	35.90	35.51	0.75	0.78
	0	0	11.2	6.41	0.37	0.71	0	0	9.82	6.40	0.48	0.67
Winter	23.02	26.37	29.66	21.51	0.84	0.88	27.05	27.71	36.78	36.16	0.74	0.76
	0	0	8.71	5.71	0.50	0.64	0	0	8.92	5.73	0.42	0.62
All	18.28	15.79	32.36	28.43	0.91	0.89	43.84	35.68	45.76	44.96	0.89	0.90
	0	0	15.35	11.46	0.58	0.72	0	0	15.66	12.37	0.53	0.64

Units are W/m^2^ for the *bias* and *RMSE*. The under line is the deseasonalized correlations.

**Figure 8:**
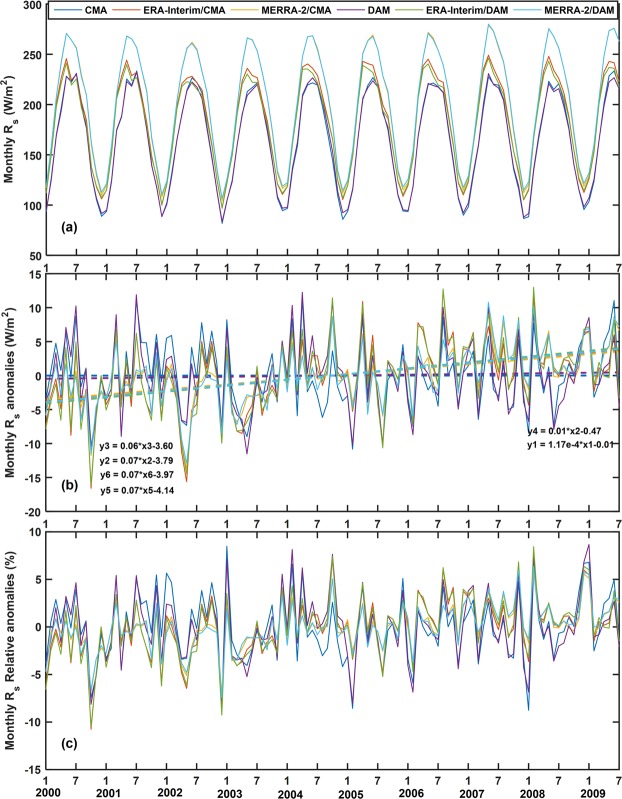
Time series of reanalysis radiation products (ERA-Interim and MERRA-2) and ground measurements from 2000 to 2009 (CMA and DAM), (**a**) monthly mean, (**b**) anomalies, (**c**) relative anomalies.

As shown in Figs. 2 and 8, the monthly mean R_s_ of the four datasets peaks around July, reaching a valley value around January every year. The monthly mean R_s_ of the datasets in annual cycle is shown in Fig. 8(a). The CMA and DAM datasets are basically fitted with a range from 80 to 230 W/m^2^. The R_s_ ranges of the ERA-Interim and MERRA-2 are 97–249 W/m^2^ and 105–280 W/m^2^, respectively. The MERRA-2 is higher than the ERA-Interim and surface measurements. The monthly mean anomalies R_s_ of the four datasets in annual cycle is shown in Fig. 8(b). The CMA and DAM datasets are basically fitted with a range from −11 to 12 W/m^2^. The R_s_ anomaly ranges of the ERA-Interim and MERRA-2 are −16–13 W/m^2^ and −13–11 W/m^2^, respectively. It can be seen that larger values of the R_s_ relative anomalies are more frequent and of larger magnitude in the MERRA-2/CMA-DAM than the ECMWF/CMA-DAM as shown in Fig. 2(c), implying that R_s_ of MERRA-2 is more changeable comparing to the ECMWF. The frequency distribution diagrams between the reanalysis and surface measurements are shown in Fig. 2(a–d). From June to October, the distribution frequency of ERA-Interim is higher with a smaller *RB* (−5 to 10%), while from December to April, the distribution frequency is higher with a larger *RB* (15 to 35%). Although the seasonal difference is not obvious in the MERRA-2, it can also be seen that the *RB* is smaller (10 to 25%) and the frequency is higher in June to October, but larger (25 to 35%) from December to April. This results are consistent with studies of Zhang *et al*.^17^ and Jia *et al*.^58^. Both ERA-Interim and MERRA-2 have lager frequency distribution with smaller *RB* in summer than winter and the difference between summer and winter is more prominent than other seasons (Fig. 2). The seasonal differences in the reanalysis products may be due to the seasonal differences in atmospheric factors mentioned in Introduction Section, which will be further analyzed in section 3.3 and 3.4.

### Bias and error assessments

**Figure 9:**
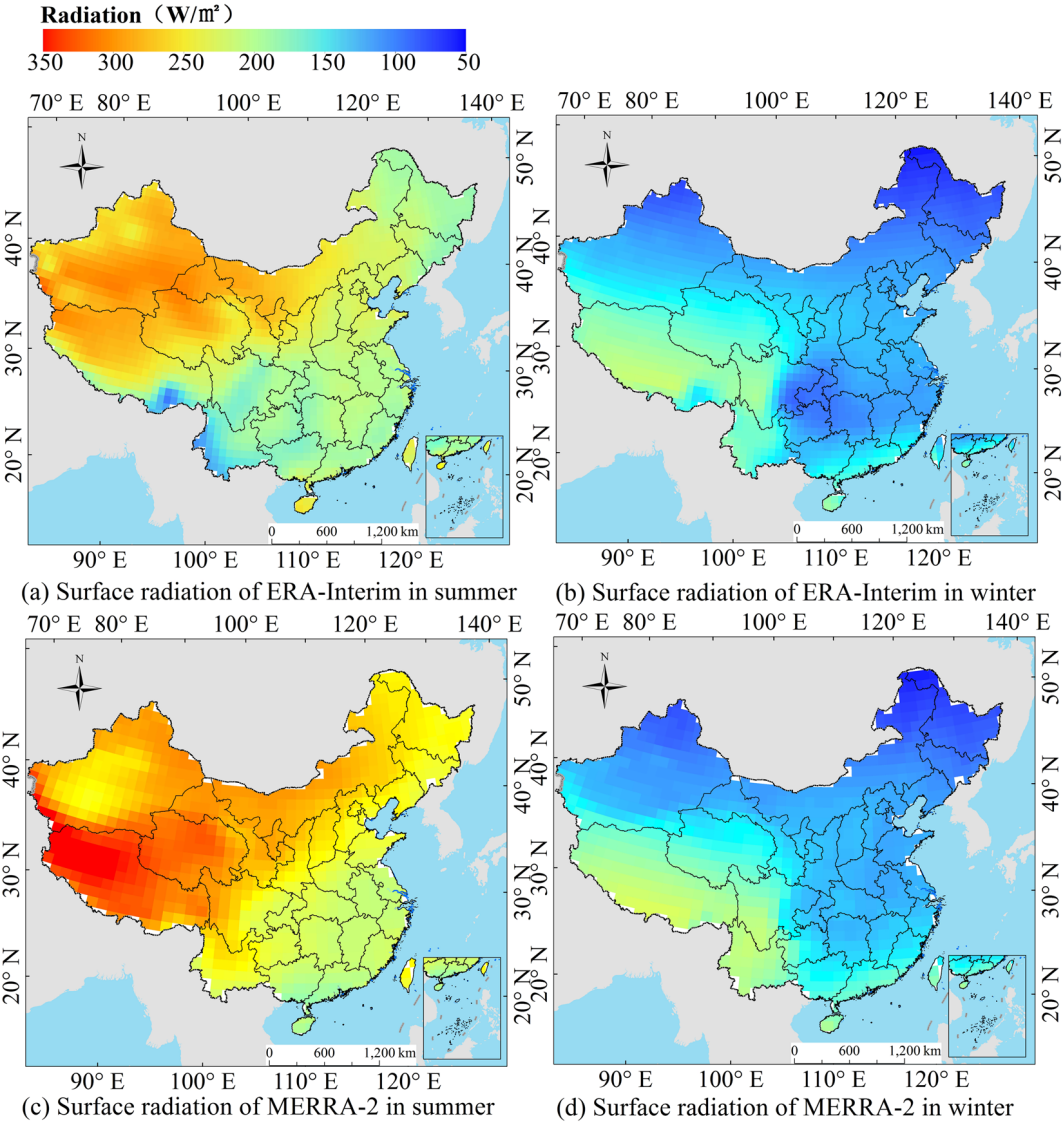
Average R_s_ distribution in the different seasons of ERA-Interim and MERRA-2 from 2000 to 2009. ((**a**,**b**) represent the radiation of ERA-Interim in summer and winter, respectively. (**c**,**d**) represent the radiation of MERRA-2 in summer and winter, respectively). (Generated by Arcgis 10.7 software, https://www.esri.com/en-us/home).

The reanalysis monthly mean products are divided into the summer and winter seasons to assess the seasonal dependency of their accuracy. Figure 9 shows the average R_s_ distribution in the different seasons of ERA-Interim and MERRA-2 from 2000 to 2009. The ranges of the radiation in summer are 100.72–309.06 W/m^2^ and 186.10–359.12 W/m^2^ for ERA-Interim and MERRA-2, respectively. The ranges in winter are 78.45–233.97 W/m^2^ and 56.52–222.53 W/m^2^, respectively. The largest R_s_ in summer is located in Qinghai, Tibet and Xinjiang. The smallest R_s_ in winter is located in Sichuan and Guizhou. The R_s_ of the MERRA-2 in summer is higher than that of the ERA-Interim in summer, while it is lower than that of the ERA-Interim in the north and Sichuan Basin in winter. The ERA-Interim and MERRA-2 basically show the same distribution characteristics of R_s_ in China, but there are obvious differences in the R_s_ values.

**Figure 10:**
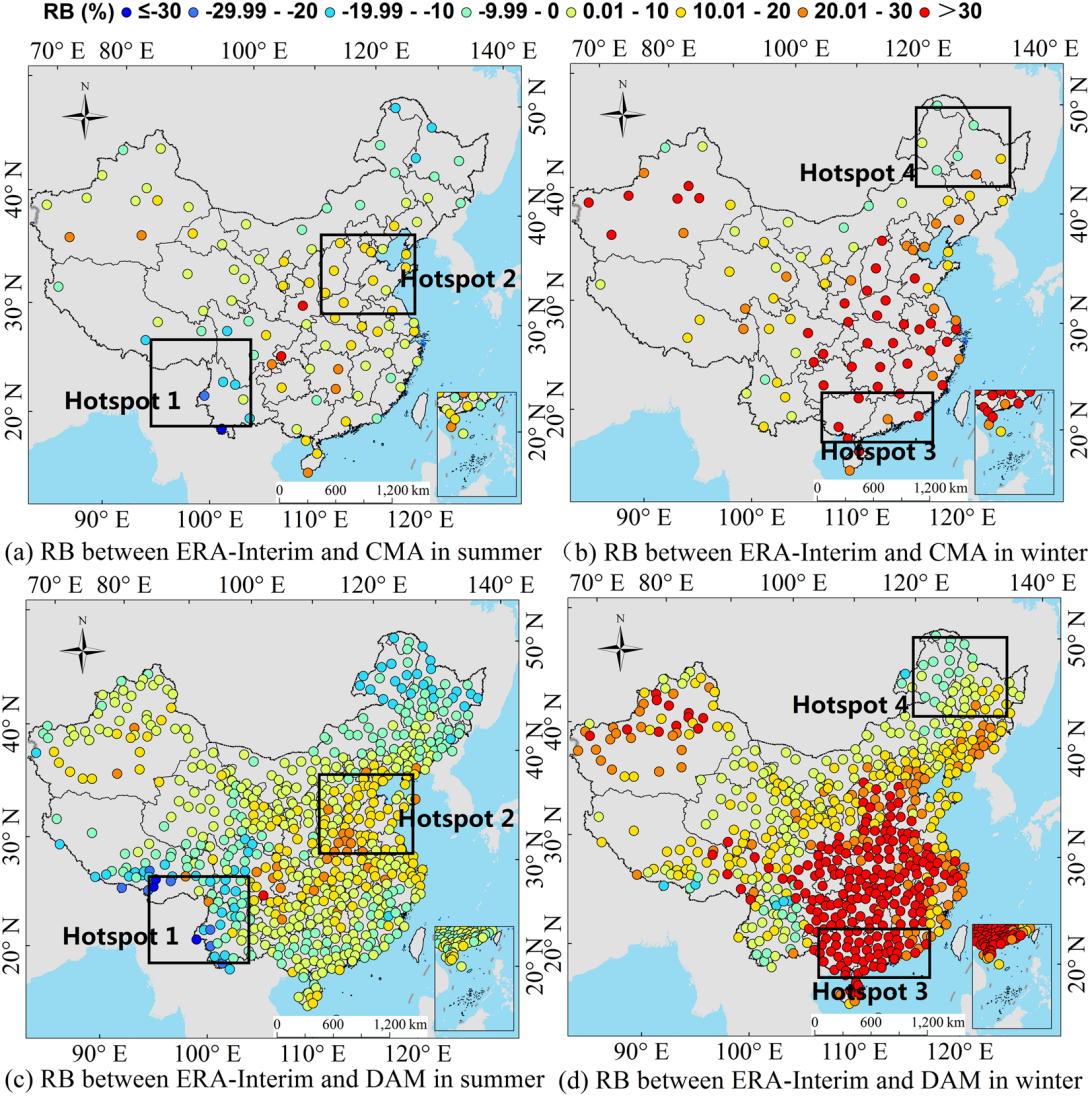
Seasonal distribution of the average R_s_ *RB* between ERA-Interim and surface measurements (CMA and DAM) from 2000 to 2009. ((**a**,**b**) represent the average radiation *RB* of ERA-Interim and CMA in summer and winter, respectively. (**c**,**d**) represent the average radiation *RB* of ERA-Interim and DAM in summer and winter, respectively.). (Generated by Arcgis 10.7 software, https://www.esri.com/en-us/home).

**Figure 11:**
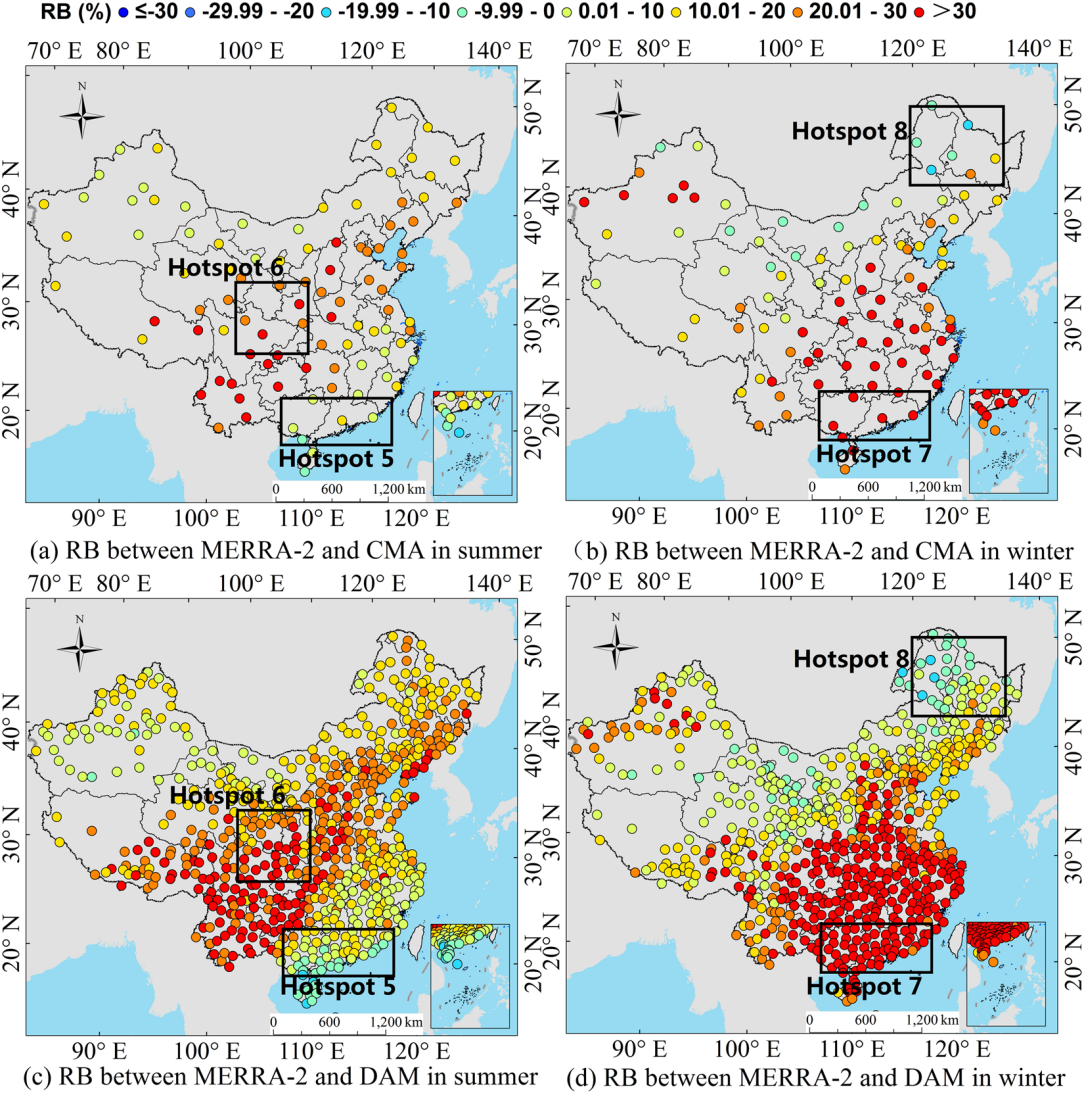
Seasonal distribution of average R_s_ *RB* between MERRA-2 and surface measurements (CMA and DAM) from 2000 to 2009. ((**a**,**b**) represent the average radiation *RB* of MERRA-2 and CMA in summer and winter, respectively. (**c**,**d**) represent the average radiation *RB* of MERRA-2 and DAM in summer and winter, respectively.). (Generated by Arcgis 10.7 software, https://www.esri.com/en-us/home).

To investigate the error distribution of the reanalysis data at the different stations, the seasonal distribution of average *RB* between ERA-Interim and surface measurements from 2000 to 2009 is shown in Figs. 10 and 11, respectively. For the ERA-Interim, in summer, the stations with positive *RB* values are mainly distributed in southeast China, central east China and the Sichuan Basin. The maximum *RB* of the CMA stations reaches 38%, and the maximum *RB* of the DAM stations reaches 35%. The stations with negative *RB* values are mainly distributed in plateaus and in southwest and northeast China. The smallest *RB* of the CMA stations is −32%, and the smallest *RB* of the DAM stations is −48%. In contrast, the *RB* is basically positive in winter, indicating that the ERA-Interim R_s_ data show overestimation. Specifically, the stations with large positive *RB* values are mainly distributed in the southeast China, central east China and the Sichuan Basin, which is the same as that in summer, and the maximum *RB* of the CMA stations is larger than 50%, and the maximum *RB* of the DAM station reaches 45%. In addition, the ERA-Interim shows seasonal differences; for example, the R_s_ in the northeast and southwest regions is underestimated during the summer and overestimated during the winter. The R_s_ in the southeast region, the central east regions and the Sichuan Basin are overestimated, but the overestimation is more pronounced in winter.

For the MERRA-2, in summer, the stations with a positive *RB* are mainly distributed in southwest China, central east China and the plateaus. The maximum *RB* of the CMA stations reaches 61%, and the maximum *RB* of DAM stations reaches 81%. The stations with a negative *RB* are mainly distributed in southeast China, especially in the coastal areas. The smallest *RB* of the CMA stations is −12%, and the smallest *RB* of the DAM stations is −17%. The errors may be affected by cloud coverage and water vapor content in the coastal areas in summer, which will be further analyzed in section 3.3 and 3.4. In winter, the *RB* is basically positive and more pronounced than in summer. The R_s_ data show overestimation. The stations with large positive *RBs* are mainly distributed in southeast and central east China. The maximum *RB* of the CMA and DAM can exceed 90%.

The data quality of reanalysis R_s_ showed regional and seasonal characteristics. The underestimation of ERA-Interim mainly occurred in the southwestern and northeastern regions. The underestimation of MERRA-2 in summer is mainly in the southern coastal region and in the northeastern region in winter. In general, both reanalysis data overestimate the R_s_ in most regions of China, and the overestimation by MERRA-2 is more obvious. The MERRA-2 and ERA-Interim both have large errors in the Sichuan Basin, possibly because of the reanalysis of atmospheric parameters, especially the influence of water vapor and cloud coverage. The further analysis will be shown in Section 3.3.

### Analysis of influence factors

The ECMWF only has MACC (Monitoring Atmospheric Composition and Climate) AOD reanalysis product, which is different from AOD used in ERA-Interim. The AOD used in the ERA-Interim radiation transfer model is Climatology AOD data, which don’t have long-term variation. So, there is no AOD product provided in the ERA-Interim, we don’t consider the impact of AOD to the R_s_ of ERA-Interim. Table 3 shows the *PD* values for each environmental factor in hotspot areas. The table header gives the names of the environmental factors: AOD, cloud coverage and water vapor content. For the ERA-Interim, in summer, the R_s_ error of the reanalysis product is mainly influenced by cloud coverage in hotspot 1 with the *PD* values of 0.4241. Although the influence of the water vapor content is small, the impact on the R_s_ could not be ignored since the *PD* value is also greater than 0.1000. Hotspot 2 is mainly affected by cloud coverage, and the *PD* value is as high as 0.2410, and the influence of water vapor content can be ignored as *PD* value (0.0912) is less than 0.1000. In winter, the dominant factors in hotspots 3 and 4 are cloud coverage, and the *PD* value of these factors is 0.3057, 0.2125, respectively. The water vapor content in hotspots 3 and 4 also passed the significance test, but the *PD* value is small. For the MERRA-2, in summer, the influence factors of hotspot 5 are ordered as follows: AOD > cloud coverage > water vapor content, and the *PD* value is 0.2452, 0.1342 and 0.1244 respectively. The influence factors of hotspot 6 are ordered as follows: AOD > cloud coverage > water vapor content, and the *PD* value is 0.2124, 0.1623 and 0.1398, respectively. In winter, Hotspots 7 and 8 are affected by AOD, cloud coverage and water vapor content, but they are mainly influenced by AOD in hotspot 7 and cloud coverage in hotspot 8. In summary, cloud coverage and AOD are the main influencing factors for the two reanalysis R_s_ products, and with the increase in cloud coverage, the influence of the water vapor content cannot be ignored.

**Table 3:** Seasonal *PD* values of the hotspot regions in China (the calculation of the influence factors is based on the DAM datasets, the numbers 1–8 represent different hotspots).

	ERA-Interim			MERRA-2			
	Hotspots	Cloud Coverage	Water Vapor	Hotspots	AOD	Cloud Coverage	Water Vapor
Summer	1	0.4241	0.1624	5	0.2452	0.1342	0.1244
	2	0.2410	0.0912	6	0.2124	0.1623	0.1398
Winter	3	0.3057	0.0895	7	0.4735	0.3823	0.1579
	4	0.2125	0.0631	8	0.2699	0.4322	0.1001

### Atmospheric parameter verification

**Figure 12:**
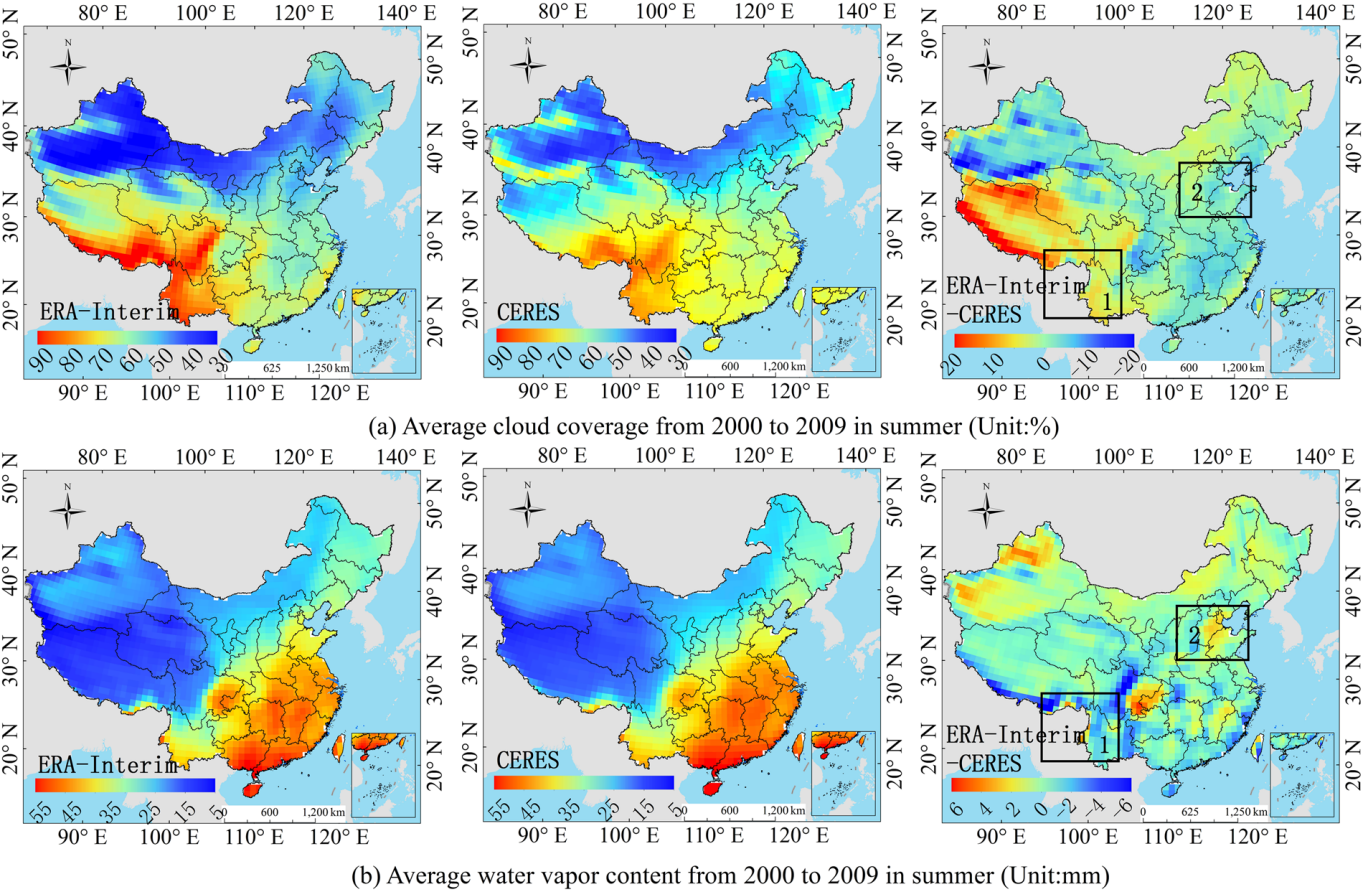
Comparison of the ERA-Interim and CERES average atmosphere product in summer on a regional scale from 2000 to 2009. ((**a**,**b**) correspond to ERA-Interim, CERES and ERA-Interim minus CERES, respectively.). (Generated by Arcgis 10.7 software, https://www.esri.com/en-us/home).

**Figure 13:**
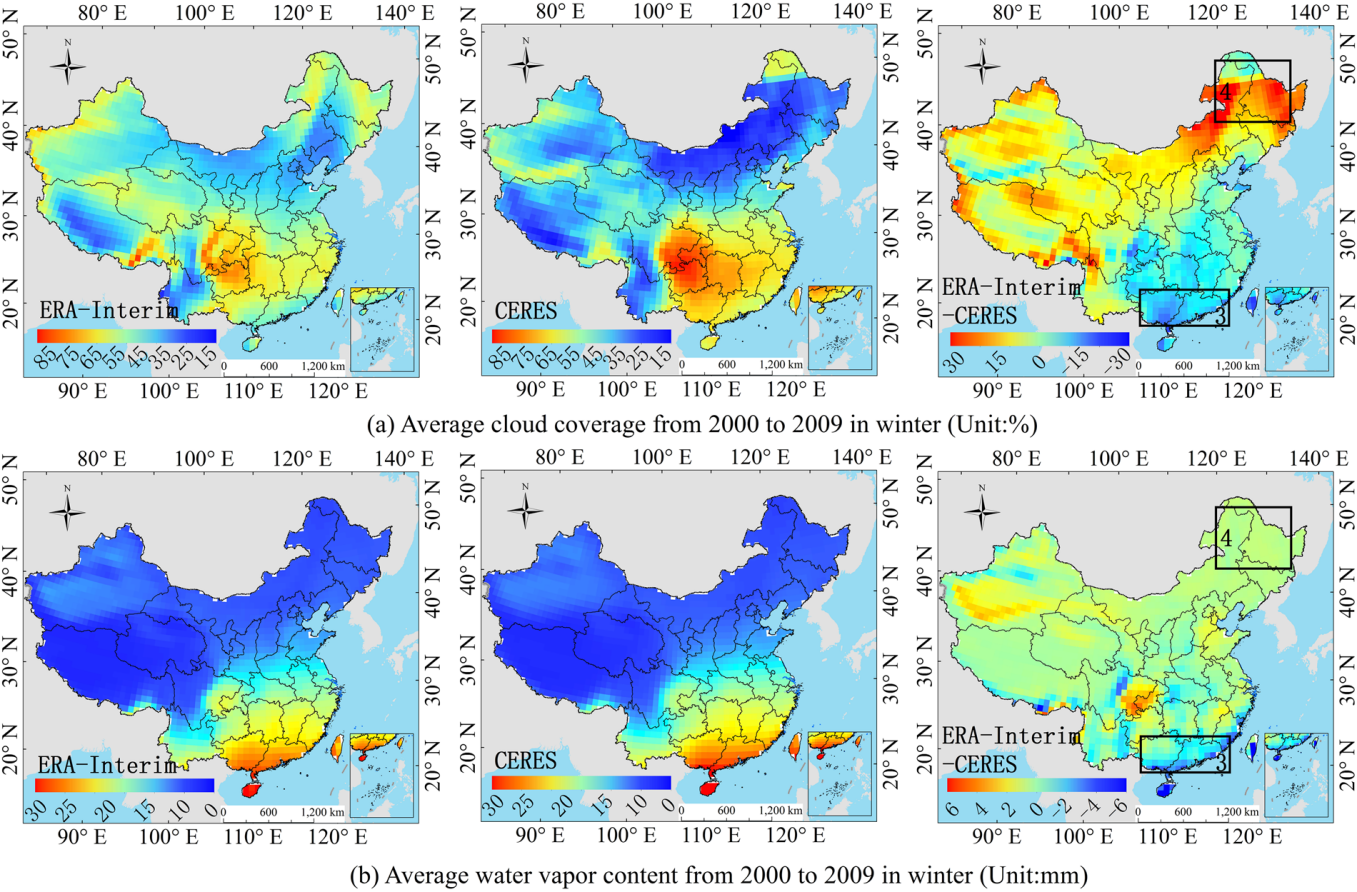
Comparison of the ERA-Interim and CERES average atmosphere product in winter on a regional scale from 2000 to 2009. ((**a**,**b**) correspond to ERA-Interim, CERES and ERA-Interim minus CERES, respectively.). (Generated by Arcgis 10.7 software, https://www.esri.com/en-us/home).

**Figure 14:**
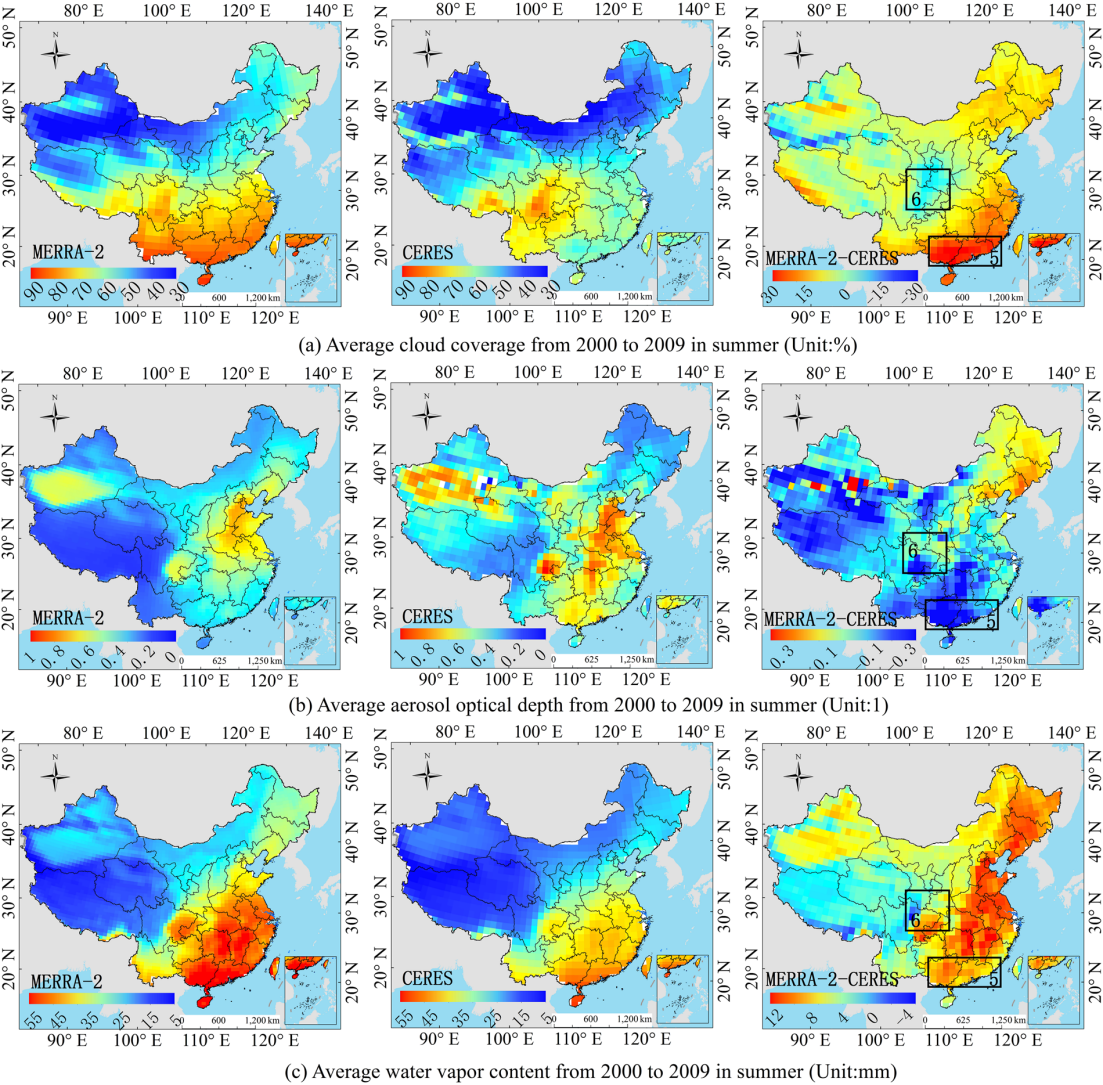
Comparison of the MERRA-2 and CERES average atmosphere product in summer on a regional scale from 2000 to 2009. ((**a**–**c**) correspond to MERRA-2, CERES and MERRA-2 minus CERES, respectively.). (Generated by Arcgis 10.7 software, https://www.esri.com/en-us/home).

**Figure 15:**
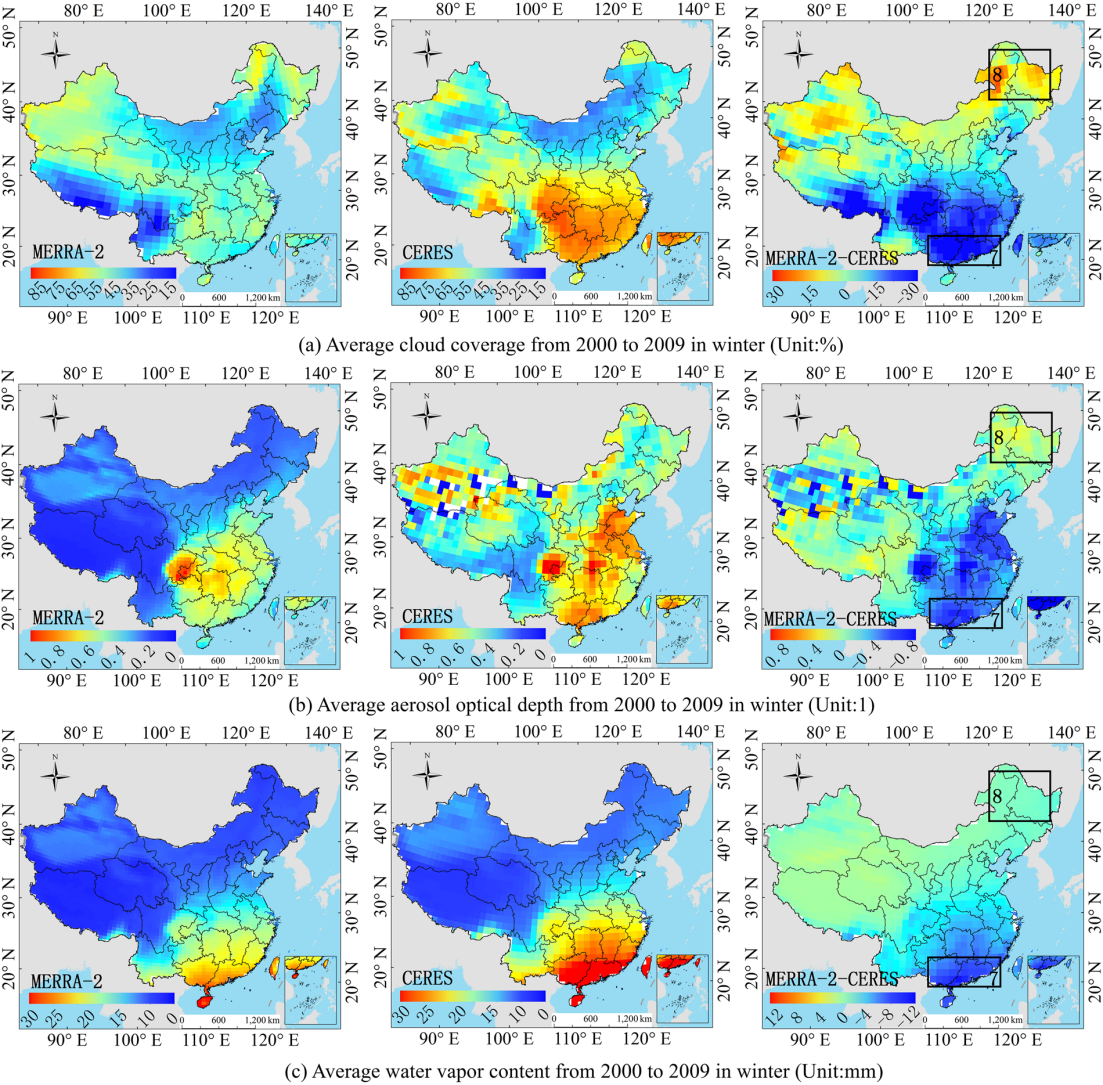
Comparison of the MERRA-2 and CERES average atmosphere product in winter on a regional scale from 2000 to 2009. ((**a**–**c**) Correspond to MERRA-2, CERES and MERRA-2 minus CERES, respectively.). (Generated by Arcgis 10.7 software, https://www.esri.com/en-us/home).

Section 3.3 explained the *PD* value of the atmospheric influence factors in each hotspot. The section determines whether the influence factors are representative and accurate. As shown in Figs. 12–15, the average cloud coverage, AOD and water vapor content of CERES-EBAF from 2000 to 2009 are compared with the ERA-Interim and MERRA-2 atmospheric products. The *PD* value (Table 3) and the deviation of the radiation (Fig. 3) in the hotspots are combined to verify whether the change in the atmospheric parameters of the reanalysis is consistent with the dominant atmospheric factors in the hotspots.

For the ERA-Interim, the R_s_ is underestimated in the hotspot 1 (Fig. 3a), and the order of influence power of atmospheric factors is cloud coverage > water vapor (Table 3). The underestimation of the water vapor only can compensate part of the underestimation of the R_s_ due to overestimation of the cloud coverage. Because cloud coverage is the dominant factor of this region (Fig. 12a), whose power is much larger than that of water vapor (Table 3). The dominant factor in hotspot 2 is the cloud coverage (Table 3), and the underestimation of cloud coverage (Fig. 12a) leads to the overestimation of the R_s_ in the hotspot 2 (Fig. 3a). It is difficult to obtain an accurate R_s_ in the southeast coastal areas (hotspot 3), because of the rapid dynamic change of the cloud cover. The results show that the dominant factors in hotspot 3 is cloud coverage (Table 3), which is underestimated (Fig. 13a), resulting in an overestimation of the ERA-Interim R_s_ in this region (Fig. 3b). In hotspot 4, the influence of cloud coverage is larger than that of other factors (Table 3). The cloud coverage is overestimated (Fig. 13a), which led to the underestimation of the R_s_ (Fig. 3b). Boilley and Wald^30^ also proved that ERA-Interim often mistakes cloudy conditions as clear skies. The opposite is also true though less pronounced: actual clear sky conditions are predicted as cloudy.

For the MERRA-2, the R_s_ is slightly underestimated in the southeast coastal areas (hotspot 5), and the order of influence power of atmospheric factors is AOD > cloud coverage > water vapor (Table 3). The underestimation of the AOD cannot compensate the underestimation of R_s_ due to the overestimation of cloud coverage and water vapor content (Fig. 14a–c), because the sum of power of cloud coverage and water vapor (*PD* = 0.2586) is greater than AOD (*PD* = 0.2452). The R_s_ in the Sichuan Basin (hotspot 6) is affected by the topography of the basin, and the estimation of the R_s_ is complicated. The influence power of AOD and cloud coverage is greater than water vapor content (Table 3). Therefore, the overestimation of water vapor content cannot compensate the overestimation of R_s_ due to the underestimation of AOD and cloud coverage (Fig. 14a,c). Therefore, the R_s_ in this region is overestimated (Fig. 3c). The dominant factors’ order in hotspot 7 is as follows: AOD > cloud coverage > water vapor content (Fig. 15a–c), and all the influence factors are obviously underestimated in this area, which led to the overestimation of the R_s_ in hotspot 7, as shown in Fig. 3d. The cloud coverage and AOD in hotspot 8 are overestimated (Fig. 15a,b), resulting in an underestimation of the R_s_ in this area. The results consistent with Feng and Wang’s^60^ study that MERRA-2 have a high mean bias over China due to their incorrect estimation of cloud fraction, which is greater in southern China. The bias in trend tend to reduce due to its underestimation of aerosol assimilation. However, MERRA-2 show a positive bias in trend of R_s_ likely due to aerosol-cloud interaction.

The study shows that the R_s_ error of the MERRA-2 and ERA-Interim in the southeast coastal areas are mainly influenced by the cloud coverage, and when the *PD* value of cloud coverage is great, the influence of water vapor content also becomes greater. The error causes in the Sichuan Basin are mainly affected by AOD and water vapor content, and those in the northeast and middle east of China is mainly affected by cloud coverage and AOD.

## Conclusions

This study presents the validation and inter-comparison of the reanalysis R_s_ estimation from 2000–2009 provided by the ERA-Interim and MERRA-2 using quality-controlled surface measurements at 96 stations from CMA and 716 R_s_ data from the DAM. The reanalysis products are also compared with the satellite retrievals of CERES-EBAF from different perspectives, including accuracy, spatial distribution, periodic variation, seasonal variation, inter-annual variation and regional variation. In addition, considering the influence of atmospheric factors on the earth’s R_s_ and the spatially stratified heterogeneity of the atmospheric distribution, the geographical detector is used to find out the errors causes in different hotspots in China.

Overall, the *bias* between the ERA-Interim products and surface measurements at all stations range from 15.79 to 18.28 W/m^2^, and the *RMSE* ranges from 28.43 to 32.36 W/m^2^. The *bias* between the MERRA-2 products and surface measurements at all stations range from 35.68 to 43.84 W/m^2^, and the *RMSE* ranges from 44.96 to 45.76 W/m^2^. Both the ERA-Interim and MERRA-2 reanalysis radiation data overestimate R_s_ in China, and the overestimation of MERRA-2 is more pronounced. The ERA-Interim has strong seasonal differences, and data in summer and autumn are better than those in spring and winter. In contrast, MERRA-2’s seasonal differences are not obvious. The study shows that R_s_ error of MERRA-2 and ERA-Interim in the southeast coastal areas are mainly influenced by cloud coverage. When the *PD* value of cloud coverage is large, the influence of water vapor content also becomes greater. The error in the Sichuan Basin is mainly affected by AOD and water vapor content, and the error in the northeast and middle east of China is mainly affected by cloud coverage and AOD.

Generally, further improvements and efforts are required for higher accuracy by using more surface measurements, and improvement of the accuracy in the atmospheric parameters would make the verification results more convincing. The results are useful for the proper application and accurate data correction about the two representative global reanalysis data. Further study will focus on establishing the empirical relationship between the factors’ *PD* values and the radiative errors of reanalysis, as well as correct the radiative errors.

## References

[1] Wen, D. M. Radiation climate in China. in *Radiation climate in China* 1–20 (China Meteorological Press, 1997).

[2] Jiang H, Lu N, Qin J, Tang W, Yao L (2019). A deep learning algorithm to estimate hourly global solar radiation from geostationary satellite data. Renew. Sustain. Energy Rev..

[3] Zhang X, Liang S, Wild M, Jiang B (2015). Analysis of surface incident shortwave radiation from four satellite products. Remote Sens. Environ..

[4] Silva José P., Balenzategui José L., Martín-Pomares Luis, Wilbert Stefan, Polo Jesús (2019). Quality Assurance of Solar Radiation Measurements. Solar Resources Mapping.

[5] Tang W, Yang K, Qin J, Min M, Niu X (2018). First Effort for Constructing a Direct Solar Radiation Data Set in China for Solar Energy Applications. J. Geophys. Res. Atmos..

[6] Ekici C, Teke I (2019). Global solar radiation estimation from measurements of visibility and air temperature extremes. Energy Sources, Part A Recover. Util. Environ. Eff..

[7] Pinker RT (2003). Surface radiation budgets in support of the GEWEX Continental-Scale International Project (GCIP) and the GEWEX Americas Prediction Project (GAPP), including the North American Land Data Assimilation System (NLDAS) project. J. Geophys. Res. D Atmos..

[8] Kato S (2013). Surface irradiances consistent with CERES-derived top-of-atmosphere shortwave and longwave irradiances. J. Clim..

[9] Simmons A, Uppala S, Dee D, Kobayashi S (2007). ERA-Interim: New ECMWF reanalysis products from 1989 onwards. ECMWF Newsl..

[10] Gelaro R (2017). The modern-era retrospective analysis for research and applications, version 2 (MERRA-2). J. Clim..

[11] Qin J (2015). An efficient physically based parameterization to derive surface solar irradiance based on satellite atmospheric products. J. Geophys. Res..

[12] Huang G, Li X, Ma M, Li H, Huang C (2016). High resolution surface radiation products for studies of regional energy, hydrologic and ecological processes over Heihe river basin, northwest China. Agric. For. Meteorol..

[13] Trenberth KE, Olson JG (1988). An Evaluation and Intercomparison of Global Analyses from the National Meteorological Center and the European Centre for Medium Range Weather Forecasts. Bull. Am. Meteorol. Soc..

[14] Betts AK (2003). Intercomparison of water and energy budgets for five Mississippi subbasins between ECMWF reanalysis (ERA-40) and NASA Data Assimilation Office fvGCM for 1990–1999. J. Geophys. Res. D Atmos..

[15] Bengtsson L, Hagemann S, Hodges KI (2004). Can climate trends be calculated from reanalysis data?. J. Geophys. Res. D Atmos..

[16] Xie X, He JH, Qi L (2011). A review on applicability evaluation of four reanalysis datasets in China. J. Meteorol. Environ..

[17] Zhang X (2016). Evaluation of the reanalysis surface incident shortwave radiation products from NCEP, ECMWF, GSFC, and JMA using satellite and surface observations. Remote Sens..

[18] Wang K, Dickinson RE (2013). Global atmospheric downward longwave radiation at the surface from ground-based observations, satellite retrievals, and reanalyses. Rev. Geophys..

[19] Decker M (2012). Evaluation of the reanalysis products from GSFC, NCEP, and ECMWF using flux tower observations. J. Clim..

[20] Lohmann S, Schillings C, Mayer B, Meyer R (2006). Long-term variability of solar direct and global radiation derived from ISCCP data and comparison with reanalysis data. Sol. Energy.

[21] Groenendijk M (2011). Assessing parameter variability in a photosynthesis model within and between plant functional types using global Fluxnet eddy covariance data. Agric. For. Meteorol..

[22] Ekici A (2014). Simulating high-latitude permafrost regions by the JSBACH terrestrial ecosystem model. Geosci. Model Dev..

[23] Kimball JS (2007). Recent climate-driven increases in vegetation productivity for the western Arctic: Evidence of an acceleration of the northern terrestrial carbon cycle. Earth Interact..

[24] Wang A, Zeng X (2012). Evaluation of multireanalysis products with *in situ* observations over the Tibetan Plateau. J. Geophys. Res. Atmos..

[25] Xia XA, Wang PC, Chen HB, Liang F (2006). Analyis of downwelling surface solar radiation in China from National Centers for Environmental Prediction reanalysis, satellite estimates, and surface observations. J. Geophys. Res. Atmos..

[26] Xingxing, Z., Ning, L., Ling, Y. & Hou, J. Error Analysis of ECMWF Surface Solar Radiation Data in China. **20**, 254–267 (2017).

[27] You Q (2013). Decadal variation of surface solar radiation in the Tibetan Plateau from observations, reanalysis and model simulations. Clim. Dyn..

[28] Yue, K. *Influence of aerosol optical depth on solar radiation in Yangtze River Delta*. (Nanjing University of Information Science and Technology, 2016).

[29] Fu L, Bian L, Xiao C (2015). Applicability evaluation of four reanalysis radiation data on the East Antarctic plateau. Polar Res..

[30] Boilley A, Wald L (2015). Comparison between meteorological re-analyses from ERA-Interim and MERRA and measurements of daily solar irradiation at surface. Renew. Energy.

[31] Penna B, Herdies D, Costa S (2018). Estimates of direct radiative forcing due to aerosols from the MERRA-2 reanalysis over the Amazon region. Atmos. Chem. Phys. Discuss..

[32] Wang J, Xu C (2017). Geodetector: Principle and prospective. Dili Xuebao/Acta Geogr. Sin..

[33] Mlawer EJ, Taubman SJ, Brown PD, Iacono MJ, Clough SA (1997). Radiative transfer for inhomogeneous atmospheres: RRTM, a validated correlated-k model for the longwave. J. Geophys. Res. D Atmos..

[34] Dee DP (2011). The ERA-Interim reanalysis: Configuration and performance of the data assimilation system. Q. J. R. Meteorol. Soc..

[35] Chou M-D, Suarez M (1999). A solar radiation parameterization (CLIRAD-SW) for atmospheric studies. NASA Tech. Memo.

[36] Ma Y, Liu X, Xu S (1998). The description of Chinese radiation data and their quality control procedures. Meteorol. Sci..

[37] Shi GY (2008). Data quality assessment and the long-term trend of ground solar radiation in China. J. Appl. Meteorol. Climatol..

[38] Yang K, Koike T, Ye B (2006). Improving estimation of hourly, daily, and monthly solar radiation by importing global data sets. Agric. For. Meteorol..

[39] Tang WJ, Yang K, Qin J, Min M (2013). Development of a 50-year daily surface solar radiation dataset over China. Sci. China Earth Sci..

[40] Tang WJ, Yang K, Qin J, Cheng CCK, He J (2011). Solar radiation trend across China in recent decades: A revisit with quality-controlled data. Atmos. Chem. Phys..

[41] Young, D. F. *et al*. Clouds and the Earth’ s Radiant Energy System (CERES) Algorithm Theoretical Basis Document ERBE-like Averaging to Monthly TOA Fluxes. **1997**, 1–26 (1997).

[42] Ma Q, Wang K, Wild M (2015). Impact of geolocations of validation data on the evaluation of surface incident shortwave radiation from earth system models. J. Geophys. Res..

[43] Li X, Wagner F, Peng W, Yang J, Mauzerall DL (2017). Reduction of solar photovoltaic resources due to air pollution in China. Proc. Natl. Acad. Sci. USA.

[44] Yan H (2015). Comparison of CERES-MODIS cloud microphysical properties with surface observations over Loess Plateau. J. Quant. Spectrosc. Radiat. Transf..

[45] Liu H, Tang S, Zhang S, Hu J (2015). Evaluation of MODIS water vapour products over China using radiosonde data. Int. J. Remote Sens..

[46] Younes S, Claywell R, Muneer T (2005). Quality control of solar radiation data: Present status and proposed new approaches. Energy.

[47] Yorukoglu M, Celik AN (2006). A critical review on the estimation of daily global solar radiation from sunshine duration. Energy Convers. Manag..

[48] Moradi I (2009). Quality control of global solar radiation using sunshine duration hours. Energy.

[49] Tang W, Yang K, He J, Qin J (2010). Quality control and estimation of global solar radiation in China. Sol. Energy.

[50] Geiger M, Diabaté L, Ménard L, Wald L (2002). A web service for controlling the quality of measurements of global solar irradiation. Sol. Energy.

[51] Zou L (2016). Long-term variations of estimated global solar radiation and the influencing factors in Hunan province, China during 1980–2013. Meteorol. Atmos. Phys..

[52] Wang L (2015). Modeling and analysis of the spatiotemporal variations of photosynthetically active radiation in China during 1961-2012. Renew. Sustain. Energy Rev..

[53] Wang L, Gong W, Hu B, Zhu Z (2014). Analysis of photosynthetically active radiation in Northwest China from observation and estimation. Int. J. Biometeorol..

[54] Lu N, Qin J, Yang K, Sun J (2011). A simple and efficient algorithm to estimate daily global solar radiation from geostationary satellite data. Energy.

[55] Wang JF (2010). Geographical detectors-based health risk assessment and its application in the neural tube defects study of the Heshun Region, China. Int. J. Geogr. Inf. Sci..

[56] Liu Y, Yang R (2012). The spatial characteristics and formation mechanism of the county urbanization in China. Dili Xuebao/Acta Geogr. Sin..

[57] Ding Y, Cai J, Ren Z (2014). Spatial differentiation and influencing factors of economic growth rate of state level economic and Technological Development Zones Based on geographer. Prog. Geogr..

[58] Jia B, Xie Z, Dai A, Shi C, Chen F (2013). Evaluation of satellite and reanalysis products of downward surface solar radiation over East Asia: Spatial and seasonal variations. J. Geophys. Res. Atmos..

[59] Yu Y (2019). Evaluation of the Himawari-8 Shortwave Downward Radiation (SWDR) Product and its Comparison With the CERES-SYN, MERRA-2, and ERA-Interim Datasets. IEEE J. Sel. Top. Appl. Earth Obs. Remote Sens..

[60] Feng F, Wang K (2019). Does the modern-era retrospective analysis for research and applications-2 aerosol reanalysis introduce an improvement in the simulation of surface solar radiation over China?. Int. J. Climatol..

